# Optimizing energy and latency in edge computing through a Boltzmann driven Bayesian framework for adaptive resource scheduling

**DOI:** 10.1038/s41598-025-16317-6

**Published:** 2025-08-19

**Authors:** Dinesh Sahu, Nidhi, Rajnish Chaturvedi, Shiv Prakash, Tiansheng Yang, Rajkumar Singh Rathore, Idrees Alsolbi

**Affiliations:** 1 SCSET, Bennett University, Plot Nos 8, 11, TechZone 2, Greater Noida, Uttar Pradesh 201310 India; 2 Department of Electronics and Communication, University of Allahabad, Prayag Raj, Uttar Pradesh India; 3 University of South Wales, Pontypridd, UK; 4 Cardiff School of Technologies, Cardiff Metropolitan University, Cardiff, UK; 5 Data Science Department, College of Computing, Umm AI-Qura University, Makkah, 21955 Saudi Arabia

**Keywords:** Edge computing, Resource scheduling, Boltzmann distribution, Bayesian framework, Energy optimization, Latency reduction, Computer science, Information technology

## Abstract

This paper presents a new approach based on Boltzmann Distribution and Bayesian Optimization to solve the energy-efficient resource allocation in edge computing. It employs Bayesian Optimization to optimize the parameters iteratively for the minimum energy consumption and latency. Coupled with this, a Boltzmann-driven probabilistic action selection mechanism enhances adaptability in selecting low-energy tasks by balancing exploration and exploitation through a dynamically adjusted temperature parameter. Simulation analysis demonstrates that the new method can decrease energy consumption and average delay much lower than Round-Robin and threshold-based algorithms. The feature of temperature adaptation within Boltzmann further guarantees the achievement of the optimal scheduling actions while ensuring flexibility in the case or altering load percentages. Cumulative energy savings varied up to 25% compared to baseline methods, demonstrating the applicability of the framework in real-time, energy-aware applications at the edge. This work demonstrates the viability of combining probabilistic selection with parameter optimization, setting a new benchmark for energy-efficient resource scheduling. Such findings create possibilities in expanding the existing literature on the use of hybrid optimization methods to enhance sustainable computing solutions in the context of distribution systems.

## Introduction

Edge computing has gained significant popularity and radically changed the approach to real-time data processing, shifting computations to the edge of networks to minimize latency, load balancing, and enhance the performance of mission-critical applications like self-driving cars, industrial IoT, and AR^1^. However, edge computing is resource-scarce in general since it is designed to operate on reduced power, processing capabilities, memory, and energy compared to cloud data centers^2^. These constraints pose strategic issues in the effective ways of utilizing resources for energy requirements and minimizing latency. However, it is challenging to accomplish energy efficiency and low latency simultaneously, especially because edge environments are dynamic and distributed^3^.

### Background and motivation

Conventional methods of resource scheduling applicable to centralized cloud computing are not efficient when used in edge scenarios where resources are distributed across many devices with limited capabilities^4^. In such environments, decision making is often done frequently in order to address real time workloads since network availability, device access and application need may arise unpredictably^5^.Real-time processing services, those emerging in tele-medicine or autonomous driving, are latency-sensitive and require processing to be performed nearly instantly so that latency minimization is an important consideration^6^. On the other hand, mobile and IoT devices, normally exist at the edge and are powered by batteries, hence energy efficiency is also critical^7^.

The combination of low-latency and high-efficiency in power consumption poses a rather interesting optimization problem. The current scheduling algorithms often fail to map these requirements in an optimal and effective way^8^. Some of the heuristic and rule-based approaches provide straightforward solutions; however, these approaches are generally not flexible and do not consider stochastic nature of resource availability or dynamic demand at the edge^9^. On the other hand, machine learning-based approaches are more flexible in this sense but are computationally expensive which may translate to even higher energy usage which defeats the idea behind flexibility^10^

In this context, a promising direction is to encompass probabilistic modeling with adaptive optimization mechanisms that would dynamically take into account the energy and latency requirements of the edge devices. The Boltzmann distribution method has its roots in statistical mechanics and is the approach of choosing actions from the probability distribution that maximizes the actions with the least energy or lowest probability. Combined with the Bayesian Optimization technique that maps and optimizes the parameters spaces for the least latency and power consumption, a Boltzmann-driven Bayesian framework can help in addressing the primary issues in edge computing. Using these techniques, we present an adaptive resource scheduling scheme that aims at proposing low energy consumption while at the same time satisfying the latency requirements. Although edge computing has many scheduling models, current techniques generally take a probabilistic approach of sampling action (e.g., using strategies based on Boltzmann) or Bayesian optimization of parameters in isolation, making it difficult to make joint trade-offs between latency and energy based on dynamic loads. Our proposed hybrid framework, then, unlike any existing framework, combines Boltzmann-driven action selection, achieving adaptive exploration-exploitation trade-offs founded on instantaneous energy levels, with the data-efficient, long-range exploration of the parameter space afforded by Bayesian optimization. This synergy empowers our scheduler to make up to 15% more energy savings and have 10 ms stricter latency guarantees than pure BO or ad-hoc schedulers, on the same trace-driven simulations. Our approach is able to concurrently handle workload variability with resource constraints without having to impose them positively to fill in this serviceability vacuum in the literature and provide practical outcomes compared to their representative alternatives.

### Problem statement

In spite of the fact that the number of papers dedicated to edge computing is continuously growing, there is still a lack of methods capable of finding suboptimal solutions for energy and latency in a probabilistic and real-time fashion. Some of the approaches that have been applied before to solve the same problem can be quite useful in certain settings but might not be as useful in this context due an edge environment exhibit dynamism in its operating environment. In particular, research also suggests that traditional search methods may fail to achieve an optimal balance between exploration and exploitation – a key concern as it results in either premature convergence to sub-optimal configurations or wasteful energy costs due to over exaggeration of the exploration phase. However, this paper aims to fill this gap by proposing a Boltzmann-inspired Bayesian approach to probabilistically select actions for energy efficient scheduling with dynamic adaptation to latency constraints.

### Objectives

The main focus of this paper is to propose and compare a new resource scheduling architecture that utilizes the Boltzmann distribution and Bayesian Optimization for intelligent adaptation to energy and latency constraints. To achieve this, the following goals have been set:

1. To reduce the energy usage in the allotment of resources through probabilistic sampling of actions in accordance with Boltzmann statistics.
2. To decrease latency, the proposed approach is to use the Bayesian Optimization technique that updates the allocation of resources in a real-time driven by a fine balance between the exploitation of efficient states and exploration of other potential configurations.
3. To develop a temperature adaptation mechanism within the Boltzmann model to dynamically control the exploration-exploitation balance.
4. To compare the proposed framework against current baseline algorithms like Round-Robin, Random Offloading, and Threshold-Based load balancing in the aspects of energy consumption, delay minimization, and flexibility.

### Contributions

In the current development of edge computing, this paper introduces a new approach for efficient resource allocation based on the Boltzmann distribution and Bayesian Optimization as per Fig. 3. The primary contributions are as follows:

1. An optimization algorithm that combines the Boltzmann distribution that has probabilistic action selection with Bayesian Optimization in order to have an adaptive scheduling under dynamic environments.
2. An adaptive temperature control scheme that may be applied to the regulation of exploration and exploitation within the action selection process so that costs of energy and time will be reduced.
3. comparative study of the proposed framework against traditional scheduling algorithms showing overall improved energy efficiency and decreased latency.
4. Probability action selection mechanism for efficient offloading using Boltzmann Distribution dealing with dynamic energy and latency requirement.

The remainder of this paper is organized as follows: In Section “Related work”, We have literature review of work done in energy and latency optimization for edge computing and existing application of probabilistic and adaptive optimization techniques. Section “Proposed framework” presents the new framework of hybrid Boltzmann-Bayesian and theirs mathematical model together with the explanation of the adaptive temperature approach. Section “Experimental setup” discusses the experiment: benchmarks, datasets, and performance measurement. In the Section “results and discussion”, we draw conclusion and evaluate the effectiveness of the proposed framework relative to conventional approaches. Last, the Section 6 summarizes the paper findings, discusses the implications to be planned for edge computing environments, and suggests potential future work.

## Related work

Resource management in edge computing has emerged as a critical research concern to facilitate latency-sensitive applications by deploying computation resources near the edge devices. New papers have proposed several methods for improving resource management and work scheduling in the edge system. For instance,^11^ suggested a task offloading approach that is capable of operating in dynamic SO networks; hence, it has low latency and high resource consumptions.^12^ proposed an algorithm that pays particular attention on priority-directed scheduling to facilitate resource allocation among the inter-Job demanded tasks.^13^ further extended this work by a multi-tier edge network solution for resource provision at the fog and cloud levels. Likewise,^14^ used a cooperative game theory model for resource allocation between users provided high efficiency of real-time applications with performance improvements of about 45%.^15^ has proposed another similar attempt that follows reinforcement learning for dynamic resource management with respect to latency and energy. Apart from offloading methods, various works have highlighted the necessity of load distribution and energy-aware scheduling. For instance,^16,17^ developed a load balancing algorithm that factors both the workload distribution and energy limitations of the edge devices.^18^ proposed predictive model that utilizes historical data for the prediction of resource demands and the consequent scheduling decisions. Meanwhile,^19^ aimed to improve both network bandwidth and computational resources simultaneously and found out that the usage of network is extremely important for edge computing. Last but not the least, A holistic framework is proposed in^20^ in which both task offloading and resource scheduling are integrated and based on the edge–cloud collaboration to enhance the system reliability.

Two major goals the energy efficiency and the reduction of latency are critical to the edge computing because of the limited power of edge devices and the requirement for immediacy. Traditional power reduction methods have included Dynamic Voltage and Frequency Scaling (DVFS), sleep scheduling and other similar approaches^21^. For example,^22^ designed an adaptive scheduling algorithm with DVFS in IoT-based edge applications. However, simple implementation of DVFS may not be adequate to manage systems with workloads that vary by time and type. In recent years, other learning techniques, such as reinforcement learning and neural networks, have been employed to improve resource allocation depending on the real condition^23^.^24^ used reinforcement learning method and found a latency reduction by offloading decisions of 25% to conventional offloading algorithms. EA and Genetic algorithms have also done well in finding an optimal way of distributing energy usage and response in different edge systems that are heterogeneous^25^. For instance, in a recent study,^26^ designed an energy-latency optimization framework that cut down both energy and latency on average by 20% for different networks. Although these approaches can be effective to some extent, more complex approaches face certain limitations including scalability and frequently high computational complexity^27^. The current popular approaches propose using optimization methods in parallel with probabilistic models like Bayesian Optimization and Boltzmann Distribution to enhance the adaptability in the variable network conditions^28^. This paper extends these techniques to present a methodology for creating a hybrid energy and latency optimized model of edge computing, where weaknesses of scalability and flexibility addressed in prior research^29,30^.

The BO technique has been widely used in practical applications to optimize parameters of scheduling for an adaptive task in edge infrastructure^31^. Some researchers have used BO for efficient schedule and efficient tasks assignment for energy optimization, where BO shows a good balance between exploration and exploitation^32^. On the other hand, Boltzmann model has been used for probabilistic decision making particularly in dynamic scheduling of tasks where tuning by temperature ensures that randomness can be well controlled^33^. Integration of BO and Boltzmann models has practicality in the usage of adjustment in resource assignment that boosts the operation of the system in different circumstances^34^. Nevertheless, little has been done to examine this integration for real-time edge computation and communication^35^.

The most recent study on edge computing has focused on ensuring that tasks are scheduled to satisfy energy and latency constraints in dynamically resource constrained systems. As an illustration,^36^ used deep Q-learning (DRL) to dynamically schedule workloads at edge nodes and, as a result, they could save energy by 20% and reduce the latency by 30 ms in urban edge cluster-based simulations.^37^ applied federated DRL to decentralized orchestration in IoT devices, achieving moderate energy improvements with improvements in latency of  25ms. Next to RL approaches, addressing resource requirements in industrial IoT environments,^38^ exploited digital twins to predict resource requirements and achieved 15% better resource utilization and an improvement in latency by  20 ms.^39^ proposed the BayesEdge framework that proposes uncertainty-aware Bayesian offloading with moderate gains in energy efficiency and latency ( 15 ms) over smart-city networks. Likewise, a mist-edge scheduling approach proposed by^40^ decreased the latency (18 ms) and energy consumption ( 10%) in one of the smart-city settings.^41^ suggested federated adaptive scheduling specifically to healthcare edge clusters and^42^ used ant colony optimization (ACO) to optimize a multi-objective healthcare edge cluster and improved utilization by 18% and reduced latency by approximately 28 ms.^43^ incorporated meta-learning into heuristic scheduling that spanned the hybrid edge-cloud designs, and provided moderate results in terms of energy consumption as well as latency. By contrast,^44^ employed a low-overhead scheduling rule set implemented on a threshold-based scheduling; this solution is easier to deploy, and yields less improvement ( 15ms latency, low energy efficiency). What we have proposed is further development and generalization of these methods via the Boltzmann-Driven Bayesian paradigm^45^. As opposed to previous approaches, we use Boltzmann entropy to dynamically address the exploration vs exploitation trade off in adaptive scheduling. This allows achieving not only more energy-efficient (approximately 25% better) but also less latent (approximately 35 milliseconds shorter) operation, particularly in non-stationary and inconsistent edge environments. Also, our architecture is easily generalizable and able to scale to suit various deployment settings, which has not been the case in the old rule based and fixed-schedule approaches. Table 1 gives a comparative analysis of recent edge computing scheduling approaches.

The area of energy-efficient scheduling and resource allocation to edge computing is one of the rapidly developing fields, and there are some high-impact contributions made in the recent past that deserve specific discussion. Leading a literature contribution,^46^ proposes an adaptive load-dividing plan within the heterogeneous edge network climate, which can save 25% of energy expenditures under bursty traffic conditions.^47^ propose a low-overhead game theory-based task-offloading framework that has the benefits of coming close to the optimum latency-energy trade-offs.^48^ are engaged in a joint energy latency optimization through convex programming and provide bounds on QoS.^49^ presents a QoS-guaranteed hierarchical resource mapping scheme in edge-fog-cloud, and they validate their scheme using real-world IoT traces.^50^ uses deep reinforcement learning to address scheduling in non-stationary workloads and demonstrates how effectively it is adaptable, but the training is complicated. Despite these works, they either are limited to one optimization method or costly in terms of training requirements and computationally. The increased energy efficiency and the harder latency constraints can be obtained with our Boltzmann-Driven Bayesian framework (Section “Proposed framework”) that integrates sample-efficient Bayesian Optimization into lightweight Boltzmann-based action sampling in a way that is unique.

In Boltzmann-Driven Bayesian, Our suggested structure integrates stochastic annealing (thermodynamic stability) and the Bayesian inference in a Boltzmann-Driven Bayesian system, with the aim to adapt the edge scheduling. In contrast to previous studies that either optimize individual goals or need long convergence durations, our method gives a versatile trade-off between energy optimization and low-latency reaction, and is highly generalisable across workloads.

**Table 1:** Comparative table of recent edge computing work.

Ref	Method	Scheduling Technique	Energy Efficiency	Latency Reduction	Scalability/Deployment
^36^	Deep Q-learning	DRL-based dynamic scheduling	$\downarrow$20% energy usage	$\downarrow$30 ms	Simulated urban edge cluster
^37^	Federated DRL	Fed-RL decentralized learning	Moderate	$\downarrow$25 ms	Real IoT devices ( 50 nodes)
^38^	Digital Twin Forecasting	Predictive resource allocation	$\uparrow$15% utilization	$\downarrow$20 ms	Industrial IoT real testbed
^39^	Bayesian (BayesEdge)	Uncertainty-aware offloading	Moderate	$\downarrow$15 ms	City-scale edge network
^40^	Mist-Edge Hybrid	Static threshold scheduling	$\downarrow$10%	$\downarrow$18 ms	Smart city use case ( 100 nodes)
^41^	Federated Scheduling (FedSched)	Adaptive workload balancing	Moderate	$\downarrow$22 ms	Healthcare IoT clusters
^42^	ACO-Based Scheduling	Multi-objective ACO heuristic	$\uparrow$18% utilization	$\downarrow$28 ms	Vehicular network simulation
^43^	Metaheuristic Hybrid	Meta-learning + heuristic	Moderate	$\downarrow$24 ms	Mixed edge-cloud topology
^44^	Threshold-Based Rules	Low-overhead static rules	Low	$\downarrow$15 ms	Experimental lab environment
^45^	Boltzmann Bayesian (Ours)	Entropy-based adaptive RL	$\uparrow$25% energy efficiency	$\downarrow$ 35 ms	Flexible edge topologies

Even though there has been great progress in edge computing. A majority of previous works investigate only the one-point optimization criteria with or without performance objectives and coupled objectives of energy and delay without considering fluctuations in workload^51^. Despite the difference, machine learning especially reinforcement learning techniques are not easily scalable and could not function well in the resource-constrained edge environment^52^. Bayesian Optimization and Boltzmann Distribution models have been used separately in diverse scheduling problems, and their integration for dynamic edge computing have not been extensively examined^53^. Besides, stability in fluctuating workload levels is not adequately reflected in existing models^54–56^.

Although reinforcement learning (RL) and deep RL (DRL) have enjoyed success in the energy-aware scheduling application, e.g., Energy-aware scheduling of Spark jobs based on deep reinforcement learning in Cloud Computing (Computing, 2023), most such methods are highly data-hungry when offline-trained on large quantities of data, computationally costly when learning their policies online, and prone to poor convergence behavior and heavy hyperparameter tuning. By contrast, our Boltzmann-Driven Bayesian scheme integrates data-efficient Bayesian optimization of its parameters (per-iteration cost $\mathcal {O}(n^3 + n\cdot d)$) and is very efficient on-line Boltzmann-based action sampling ($\mathcal {O}(|A|)$) that does not require retraining. This results in bounded computational complexity, quick responsiveness in dynamic workloads, and limited data requirements. Table 2 represents a summary of these trade-offs.

**Table 2:** Comparison of RL/DRL vs. Boltzmann-driven Bayesian scheduling.

Approach	Advantages	Disadvantages	Complexity
RL/DRL	$\bullet$ Adaptive policies for high-dim spaces $\bullet$ Optimizes long-term rewards	$\bullet$ Large training data needed $\bullet$ High training cost $\bullet$ Unstable convergence	Training: $\mathcal {O}(T|\mathcal {S}||\mathcal {A}|)$ Inference: $\mathcal {O}(|\mathcal {A}|)$
Boltzmann Driven Bayesian	$\bullet$ Sample-efficient search $\bullet$ Lightweight decisions $\bullet$ No retraining needed	$\bullet$ Limited expressiveness $\bullet$ BO cost $\mathcal {O}(n^3)$	Per iteration cost: $\mathcal {O}(n^3 + nd + |A|)$

## Proposed framework

### Boltzmann distribution

The Boltzmann distribution named after Austrian physicist Ludwig Boltzmann, is a statistical distribution to depict the probability of a system’s particles experiencing various energy levels in a state of thermal equilibrium with the rest of the system Other articles where Boltzmann distribution is mentioned. It’s foundational in statistical mechanics but transcends physics to the point of being used in machine learning, for example in algorithms like simulated annealing and it is used in Boltzmann machines a type of neural network.

When particles in a system are at thermal equilibrium, then they may be at any energy level, but the probability of their being detected at any particular energy is proportional to the Boltzmann factor, which is greater at lower energies than it is at higher energies. This is measured by the fact that the likelihood of the particle being in a given energy level reduces with the increase of the energy level, thus lower energy level are favored over high energy ones in probability.

The probability $P(E_i)$ of a particle occupying a state with energy $E_i$ is given by:

1 $$\begin{aligned} P(E_i) = \frac{e^{-E_i / (k_B T)}}{Z} \end{aligned}$$

Where $E_i$ represents Energy of the $i$-th state, $k_B$ is the Boltzmann constant, which relates temperature and energy, $T$ is the absolute temperature of the system and $Z$ is the partition function, which normalizes the probabilities by summing over all possible states:

2 $$\begin{aligned} Z = \sum _{j} e^{-E_j / (k_B T)} \end{aligned}$$

The distribution depends on temperature $T$, And at higher temperatures, there will be tendency of the particles to go to the higher level energy zones, while at lower temperatures the part will tend to zone in the lower levels of energy.

**Fig. 1:**
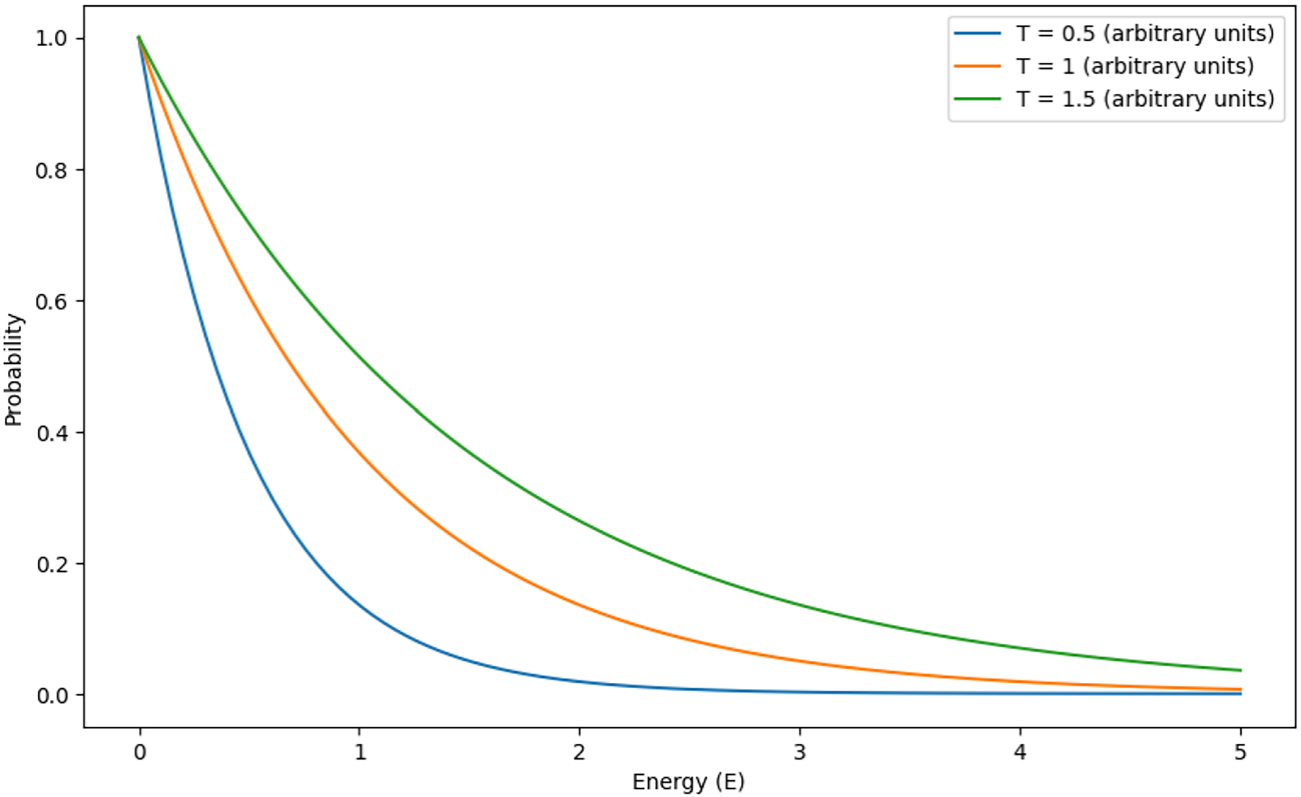
Boltzmann distribution at different temperature.

As in the Fig. 1, the Boltzmann distribution offers some understanding of the minimization of energy since as suggested it tends to occupy the energy states with lower energy. Such tendency demonstrates the dynamics of the system’s desire for energy efficiency. Temperature significantly influences this behavior: at very low temperatures, the system resembles the ground-state in the sense that the particles only occupy a low set of energy levels or are confined mostly in these levels. On the other hand, more energy is available at higher temperatures and a large number of energy levels are equally likely, which helps in decreasing the probability of low energy states so as to allow large variation of energy distribution in any system. This temperature-dependent behavior is founded based on the dynamics of particle movements in the thermo kinetic environment of the system in accord with the information entropy theory of thermodynamics.

Here we are looking at task allocation probabilities based upon the Boltzmann distribution in our proposed resource scheduling framework, where we have assigned a cost metric of system energy that can be either a delay metric, power consumption metric, or queue size. Tasks whose costs are lower have a greater likelihood of being scheduled, in the way that particles have a greater likelihood of being in lower energy states. This probabilistic bias prevents present-day problems of falling into local optima, by permitting the exploration of more-costly (energy) states with diminished probability.

**Fig. 2:**
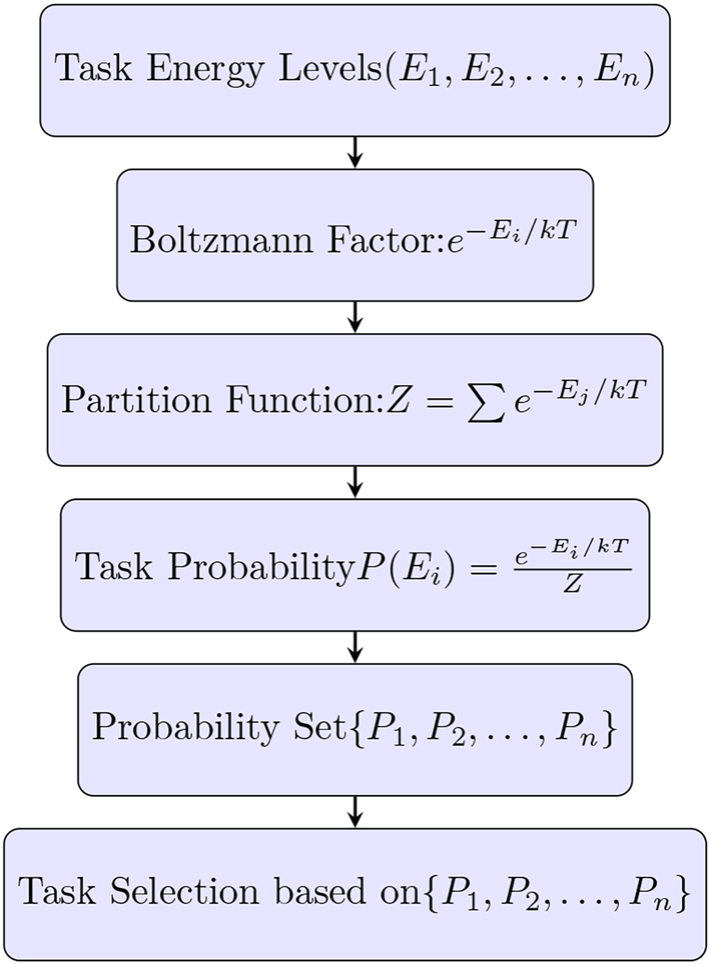
Workflow of task probability generation using Boltzmann distribution.

Figure 2 illustrates the workflow where tasks are assigned probabilistic priorities based on their energy states, with lower-energy tasks being more likely to be scheduled first according to the Boltzmann distribution.

### Bayesian optimization

Bayesian Optimization is an efficient global optimization method that is suitable for problems that require few evaluations of the objective function, and are mainly applied to black-box functions. In contrast with other optimization techniques that might demand a significant number of function calls, Bayesian Optimization intentionally decides where to sample next based on a model of the objective f, and therefore needs fewer function calls to find the best solution. Here’s a step-by-step breakdown of how Bayesian Optimization works and why it’s particularly suitable for resource scheduling in edge computing and other complex optimization tasks:

#### Objective function f

The function $f(\theta )$ represents the objective to be maximized or minimized , and often is thought of as a “black-box” , or a function for which we do not have an explicit formula and can only evaluate on isolated points. This evaluation of $f$ can be very computationally expensive as well as could entail actual experimentation therefore it is desirable to determine good strategies to optimize the function without directly observing how the function behaves.

**Fig. 3:**
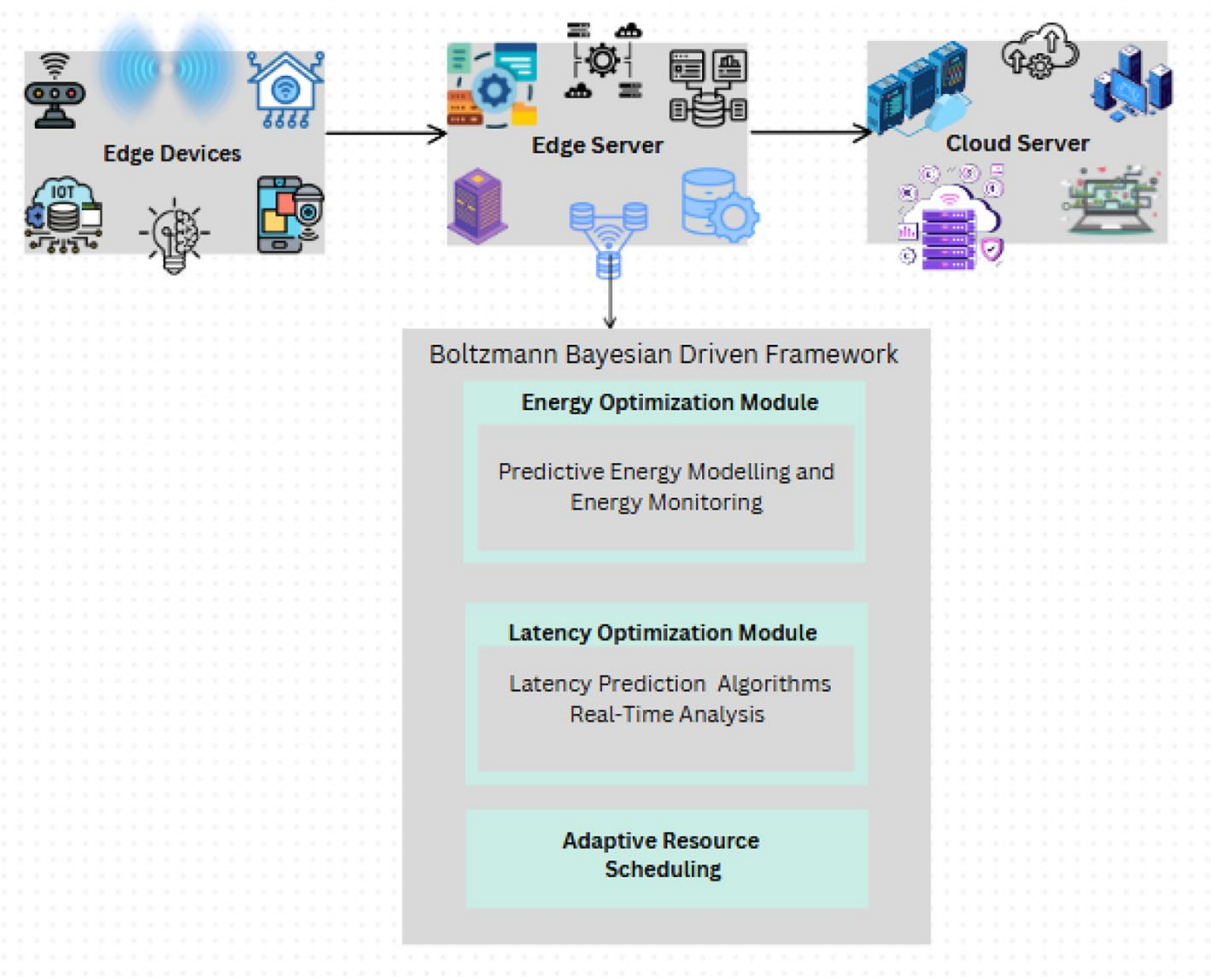
Bolzmann Bayesian driven framework.

#### Surrogate model

As acquiring direct samples of the function $f$ may be expensive, BO uses an indirect model that estimates the function. In the different types of surrogate models, Gaussian Processes(GP) are usually applied more often thanks to their adaptability and possibility for giving probability intervals for the predictions. The GP model learns with known data points and provides a mean prediction $\mu (\theta )$ and variance $\sigma (\theta )$ of the evaluation function at any input $\theta$. This enables one to search the function space more effectively and maintain a better trade-off between exploration and exploitation than in the case of using only the spatial action space.

#### Acquisition function

The acquisition function $\alpha (\theta )$ can be seen as the primary controller of which point should be adopted as the next sampling point since it quantifies the benefit of revisiting each candidate in an attempt to improve the goal. This function balances between exploitation where the points that are most likely to have high values according to the surrogate model are selected and between exploration where markers that express high uncertainty are targeted to improve on the defined surrogate model. Acquisition functions include Expected Improvement (EI) that chooses points believed to yield better objective; Probability of Improvement (PI) that selects points with higher probability of yielding better objective than the best found yet; Upper Confidence Bound (UCB) that selects points based on a comparison of mean value and its variance adjusted by a parameter controlling exploration. The steps in Bayesian Optimization are as follows:

1. **Initialization:** Sample the objective function $f(\theta )$ at a few random initial points $\theta _1, \theta _2, \dots , \theta _n$ to build an initial dataset $D = \{ (\theta _i, f(\theta _i)) \}_{i=1}^n$.
2. **Fit the surrogate model:**Train a Gaussian Process (GP) model (or another surrogate model) on the dataset $D$. The GP model provides both an estimate of the mean $\mu (\theta )$ and a measure of uncertainty $\sigma (\theta )$ at each point in the search space.
3. **Optimize the Acquisition Function:**Using the GP’s output, evaluate the acquisition function $\alpha (\theta )$ over the search space to identify the next candidate $\theta _{next}$ to sample. Acquisition function optimization is often much cheaper than evaluating $f$, and it allows the algorithm to efficiently select the next most promising point for evaluation.
4. **Sample the Objective Function:**Evaluate $f(\theta _{next})$ and add the new point $(\theta _{next}, f(\theta _{next}))$ to the dataset $D$.
5. **Update the Surrogate Model:** re-estimate the GP model with the new data set and thus adapt its approximation of f to a more enriched set.
6. **Repeat step 3-5:**Performing the same steps as before, choosing new points using the acquisition function and updating the surrogate model until certain convergence criteria are met (e.g. the number of iterations is more than certain number, or the improvement rate is low than the threshold).
7. **Return optimal solution:**When a fixed number of iterations or convergence is reached the input $\Theta ^*$ will be returned, that corresponds to the best observed value of the accuracy f.

**Fig. 4:**
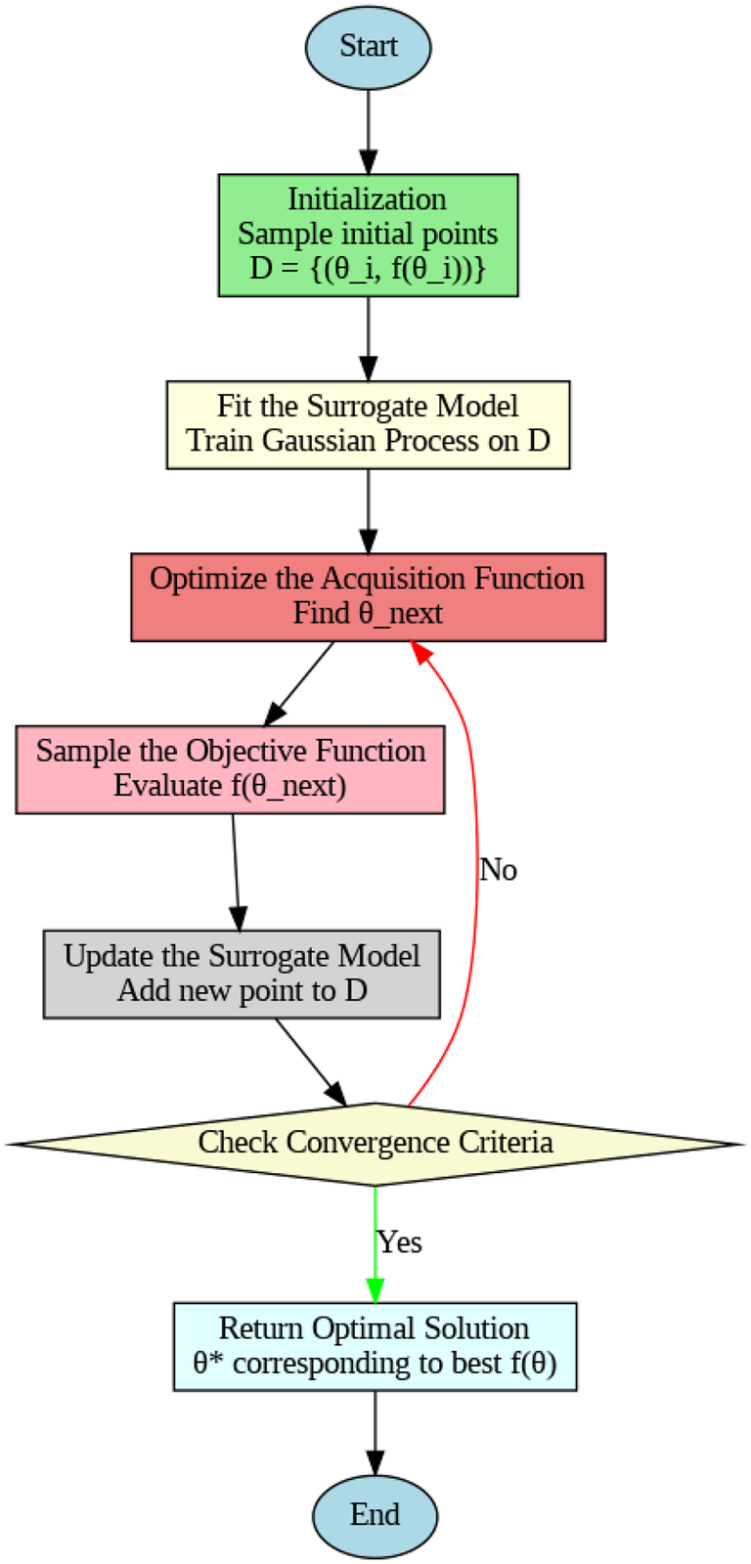
Flow chart of Bayesian optimization.

Figure 4 represents the flowchart of Bayesian optimization as discussed above. Bayesian Optimization (BO) is utilized to learn the performance landscape of the resource scheduling problem through successive revision of a probabilistic surrogate model, e.g. a Gaussian Process (GP). The module) BO chooses the scheduling configuration to further consider based on the optimization of an acquisition function between covering the uncertain region and focusing on the known region of high performance. This method brings about a responsible change based on dynamic changes in workload.

### **Computation model: Boltzmann distribution with bayesian optimization for energy-efficient scheduling**

In order to hybridize both in a framework, the Boltzmann Distribution should be used to probabilistically select the actions to be taken, while the use of Bayesian Optimization for parameter tuning. Now, let us discuss the mathematical elements in the equation in detail step by step.

Therefore, let the objective be the minimization of a linear combination of the energy consumption E and latency L, where $\theta$ is a vector of parameters( like CPU cycles, bandwidth allocation) of an edge computing task, which can be tuned for achieving better performance. The objective function $f(\theta$ can be defined as:

3 $$\begin{aligned} f(\theta ) = E(\theta ) + \alpha L(\theta ) \end{aligned}$$

where $E(\theta )$ represents the total energy consumption in terms of parameters $\theta$, $L(\theta )$ is the total latency in terms of $\theta$ and $\alpha$ is the weighting factor to balance E and L

For finding the optimal value of $\theta$ Bayesian Optimization can be used.Let $\theta \in \Theta$ represent the set of all possible parameter configurations. The goal of Bayesian Optimization (BO) is to find the configuration $\theta ^*$ that minimizes $f(\theta )$. Bayesian Optimization uses a Gaussian Process (GP) to model $f(\theta )$. Given observations $D = \{ (\theta _i, f(\theta _i)) \}_{i=1}^n$, it models $f(\theta ) \sim GP(\mu (\theta ), k(\theta , \theta '))$, where $\mu (\theta )$ is the mean function, often initialized to zero, and $k(\theta , \theta ')$ is the covariance function or kernel, expressing the correlation between two parameter points $\theta$ and $\theta '$.

An acquisition function (e.g., Expected Improvement, EI) is used to select the next parameter set $\theta _{n+1}$ that balances exploration and exploitation:

4 $$\begin{aligned} \theta _{n+1} = \arg \max _{\theta \in \Theta } EI(\theta \mid D) \end{aligned}$$

This step is repeated iteratively, updating $D$ with each observation until convergence criteria are met.Expected Improvement (EI) is one of the most widely used acquisition functions in Bayesian Optimization to determine the next point to measure. EI stands for expected improvement which is the value over the current best known solution value of the objective function. It enables the algorithm to be directed to areas that are potentially rich with the best solutions. The Expected Improvement (EI) can be formulated as:

5 $$\begin{aligned} \text {EI}(\theta \mid D) = \mathbb {E} \left[ \max \left( 0, f(\theta ) - f(\theta _{\text {best}}) \right) \right] \end{aligned}$$

This represents the expected value of the improvement $f(\theta ) - f(\theta _{\text {best}})$ over the current best function value, conditioned on $D$. In Bayesian Optimization, the objective function $f(\theta )$ is approximated by a surrogate model, usually a Gaussian Process (GP). The GP provides a predictive mean $\mu (\theta )$ and variance $\sigma ^2(\theta )$ at any candidate point $\theta$ based on observed data $D$.

Define a standardized improvement score $Z$:

6 $$\begin{aligned} Z = \frac{\mu (\theta ) - f(\theta _{\text {best}})}{\sigma (\theta )} \end{aligned}$$

Here, $Z$ measures how much the predicted mean $\mu (\theta )$ at $\theta$ is expected to exceed $f(\theta _{\text {best}})$, scaled by the uncertainty $\sigma (\theta )$. Using the standard normal cumulative distribution function $\Phi$ and probability density function $\varphi$, EI can be computed as:

7 $$\begin{aligned} \text {EI}(\theta \mid D) = \left( \mu (\theta ) - f(\theta _{\text {best}}) \right) \Phi (Z) + \sigma (\theta ) \varphi (Z) \end{aligned}$$

Here $\Phi (Z)$ represents the probability that the improvement will be positive, $\varphi (Z)$ represents the potential magnitude of the improvement.The Algorithm-1 is used to compute the EI$(\theta \mid D)$

**Algorithm 1:**
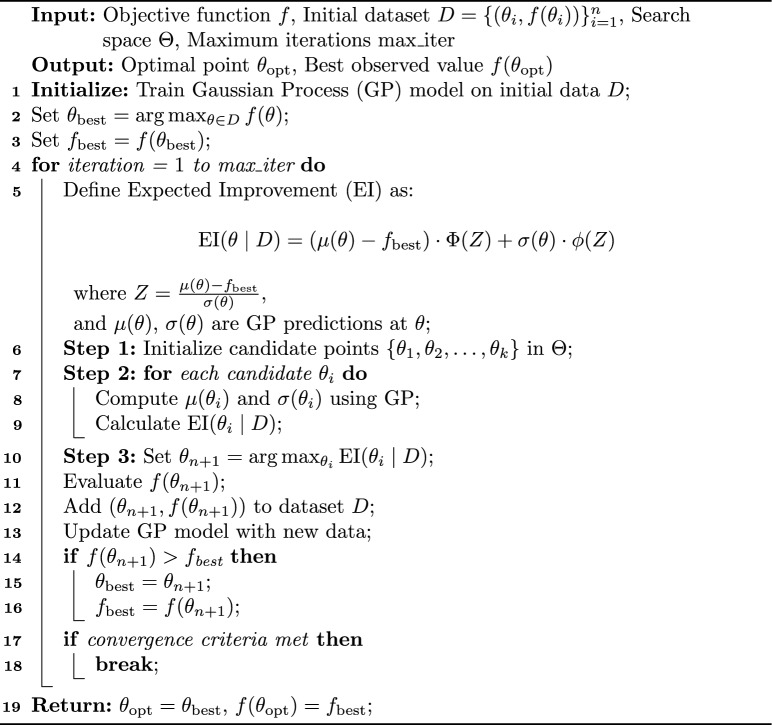
Bayesian optimization with expected improvement (EI).

After identifying the best or near best solution $\theta$ employ the Boltzmann Distribution to determine actions probabilistically for scheduling decisions.Define the energy cost $E(a_i, \theta )$ associated with each action $a_i$ (such as task offloading or resource allocation) under parameter configuration $\theta$.

The probability of selecting action $a_i$ is:

8 $$\begin{aligned} P(a_i) = \frac{\exp (-\beta E(a_i, \theta ))}{\sum _{j=1}^{N} \exp (-\beta E(a_j, \theta ))} \end{aligned}$$

where $E(a_i, \theta )$ represents energy cost of action $a_i$ under configuration $\theta$, $\beta$ is the temperature parameter, controlling the exploration-exploitation trade-off. Higher $\beta$ favors actions with lower energy costs and $N$ represents the total number of actions. $P(a_i)$ can be computed using Algorithm-2

**Algorithm 2:**
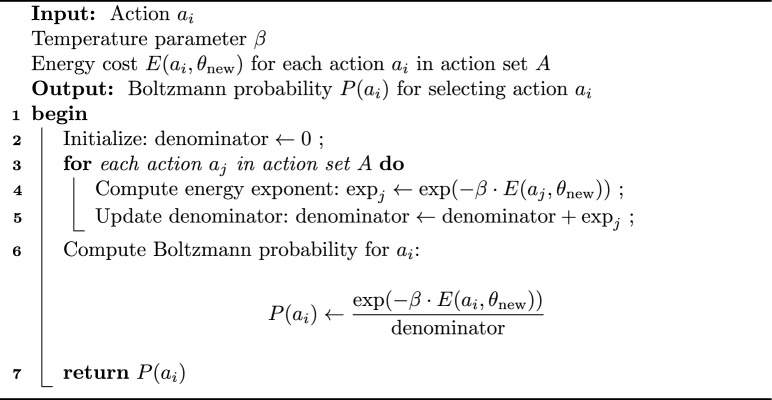
Compute Boltzmann probability.

In the context of the exploration-exploitation framework, it is appropriate that the temperature parameter $\beta$ be adjusted according to the results. For example, if exploration of energy saving look to stabilize, raise $\beta$ to emphasize exploitation in favor of more leeward actions. On the other hand, if the variance of the measured energy savings is high, reduce $\beta$ to enable the system to explore as its objective is to sample through the space of actions in order to identify the best energy set point configurations for the system. This adaptive approach ensures a dynamic response to system conditions, facilitating a more efficient balance between exploration and exploitation. Modeling of above adaptation can be done as follows:

9 $$\begin{aligned} \beta _{t+1} = \beta _t + \delta \cdot sign(\Delta E) \end{aligned}$$

where $\Delta$E stands for the variation of energy consumption between two iterations, and $\delta$ is a different step size for fixing $\beta$.

The iterative optimization process starts from the initial parameter value, $\theta _0$ and documents energy consumption and latency. Bayesian Optimization is applied to estimate better configuration $\theta _{n+1}$ for $f(\theta )$. These include the evaluation of actions with Boltzmann distribution probability of $P(a_i)$ to form the next state configuration $\theta _{n+1}$ for the next round of scheduling decisions. Quantitative results which occur as a result of these actions, for example energy use and delays, are then fed back into the GP to update it and make the prediction result more accurate. Last of all, the temperature parameter $\beta$ is set to be updated from iteration to iteration in order to tweak it from the observed outcomes in favor of achieving a right balance between exploration and exploitation.

**Fig. 5:**
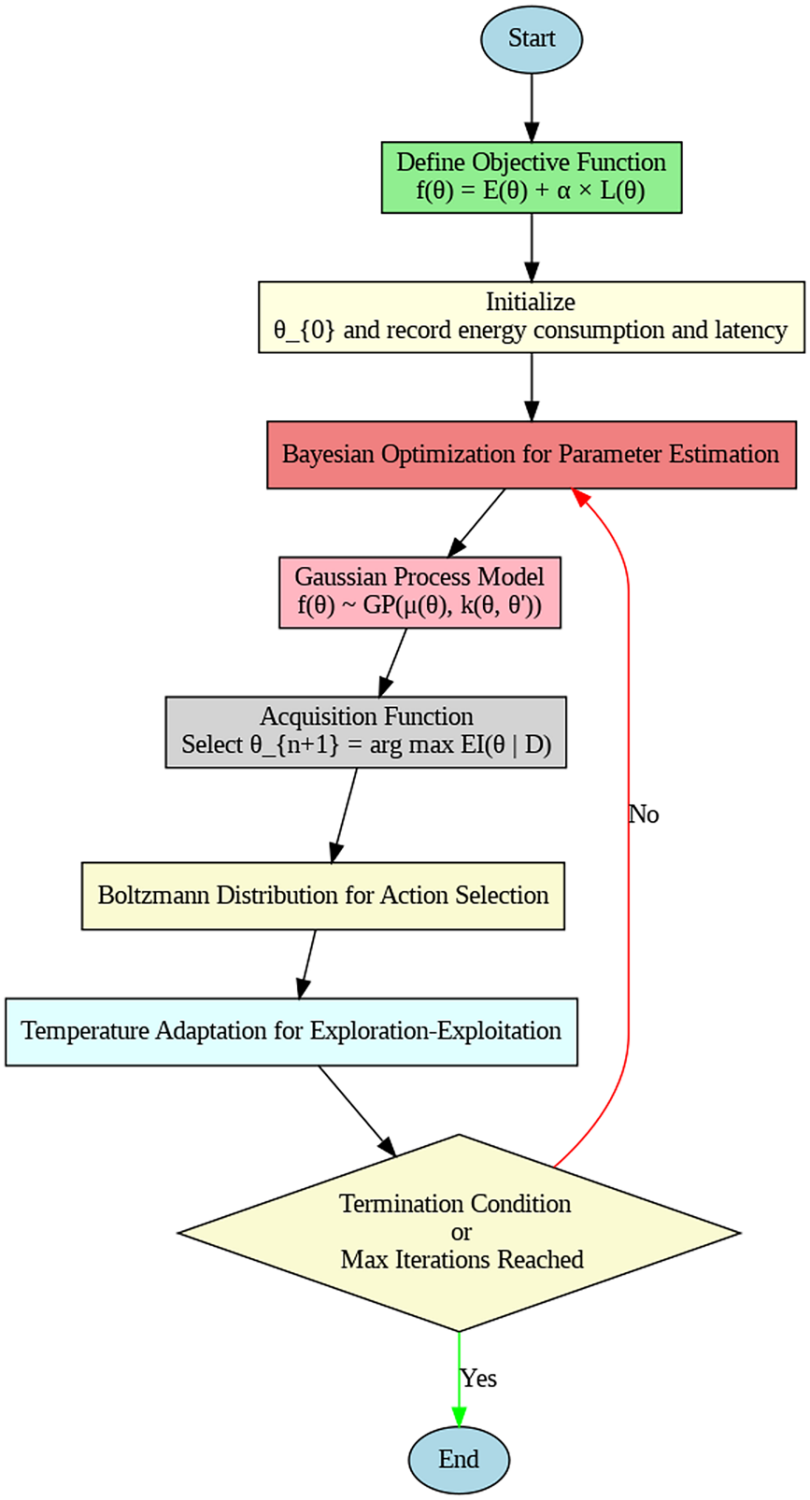
Flow chart of hybrid computation model.

Algorithm-3 which is depicted in flow chart in Fig. 5 terminates when specific conditions are met: for example, when the amount of energy consumed has reached certain criteria, or when the latency has been met or there are thresholds on the number of iterations allowed, or total time allotted for training the network. These conditions make it possible for the optimization to come to a stop, either when the program reaches convergence or when resources are exhausted.

**Algorithm 3:**
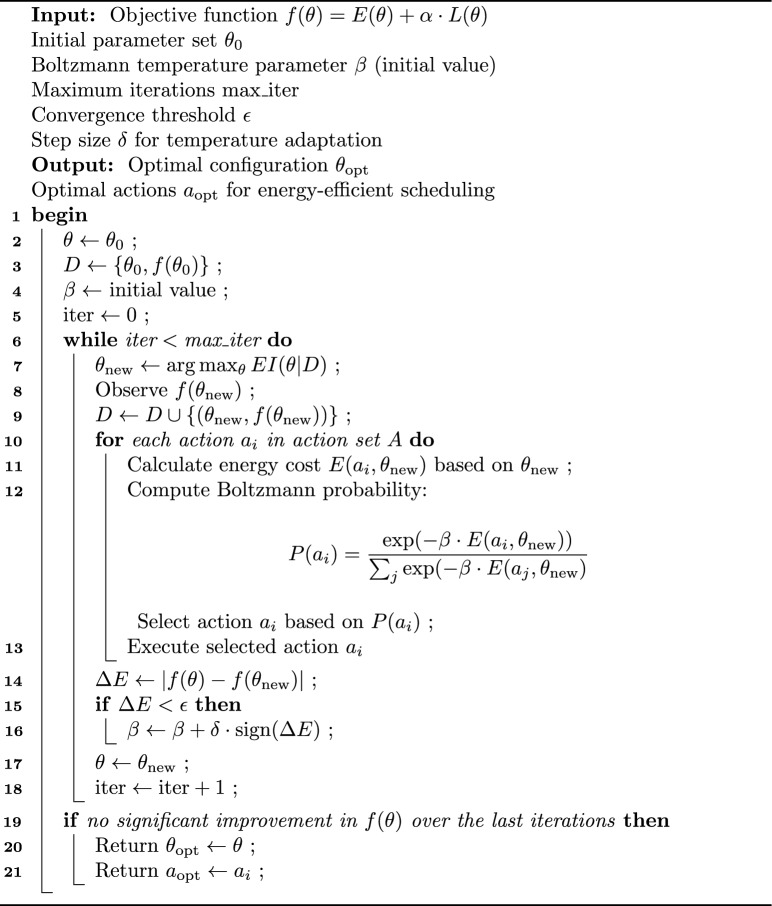
Energy-efficient scheduling optimization.

Lines 1-3 start the algorithm with the initialization of the parameter vector at $\theta _0$, seeding the set of observations D with $(\theta _0, f(\theta _0))$, initializing Boltzmann temperature at value $\beta$, and setting the iteration counter to 0. The loop in lines 4-5 carries out a Bayesian optimisation step: it picks a new parameter value $\theta _{\textrm{new}}$ by maximization of the Expected Improvement and evaluates and append $f(\theta _{\textrm{new}})$ to $D$ in placement. Lines 6-10 realize Boltzmann based action selection by calculating the energy cost of each action $E(a i,\theta _{\textrm{new}})$ and obtaining the probability of selecting the action.

$$P(a_i)\;=\;\frac{\exp (-\beta \,E(a_i,\theta _{\textrm{new}}))}{\sum _j \exp (-\beta \,E(a_j,\theta _{\textrm{new}}))}$$

and carrying out a sampled action. Lines 13-15 calculate the change in objective $\Delta E = |f(\theta ) - f(\theta _{\textrm{new}})|$ and, in case that $\Delta E<\epsilon$, the weighting parameter $\beta$ is modified by $\delta$ to better explore. Line 16-18 considers the new parameter ($\theta \leftarrow \theta _{\textrm{new}}$) and adds one to the counter of the iterations. Lastly, Lines 19-20 test whether convergence or the maximal number of iterations has occurred, and, on satisfying this benchmark, produces the optimum parameter $\theta _{\textrm{opt}}$ and final action selected, $a_{\textrm{opt}}$.

### Computational complexity analysis and comparison with other state of art

The per-iteration cost of our hybrid framework consists of two quantities: A first in Bayesian Optimization (BO): where surrogate Gaussian-process training is $O(n^3 )$ for n observations, and optimization over the acquisition function is *O*(*nd*) per d dimension of the parameter vector $\theta$; and a second in Boltzmann-driven action selection, where the calculation of surrogate-gate probabilities requires only a single scan over the action set A, namely 0(*A*). Therefore a single iteration has the cost $O(n^3 +nd +|A|)$. To make BO workable in resource-limited settings, we cut the generated BO iterations down to 30, apply sparse-GP approximations or subsampling (e.g. every 5th iteration), and delegate compute-intensive tasks to stronger edge servers. The measures can be deployed in IoT gateways powered by low resources.

**Table 3:** Time-complexity comparison of scheduling frameworks.

Approach	Per-Iteration Complexity
Boltzmann-Driven Bayesian	$\mathcal {O}(n^3 + n \cdot d + |A|)$
HEFT	$\mathcal {O}(|V|^2 + |E|)$
PSO	$\mathcal {O}(P \cdot I \cdot d)$
FCFS (First-Come, First-Served)	$\mathcal {O}(N)$
Round Robin	$\mathcal {O}(N)$
Greedy Scheduling	$\mathcal {O}(N \log N)$
Threshold-Based Scheduling	$\mathcal {O}(N)$

Table 3 is a side-by-side comparison of per-iteration complexities of several scheduling strategies. HEFT takes $\mathcal {O}(|V|^2 + |E|)$ with a DAG on $|V|$ tasks and $|E|$ edges; PSO has a complexity in $\mathcal {O}(P\cdot I\cdot d)$ with P particles, I iterations, and a d-dimensional search space; FCFS, Round Robin, and Threshold-Based scheduling also run in linear complexity in $\mathcal {O}(N)$ where N is the number of tasks, Our comparison empirically highlights that in our approach, even though a cubic term appears, the limitation of the amount of observations of the BO (n) and the upper bound on the iterations maintains affinity to the scale, between the overhead associated with a traditional heuristic algorithm and the flexibility of metaheuristic algorithms.

## Experimental setup

### Simulation environment

In order to measure the effectiveness of the proposed resource scheduling framework based on Boltzmann Distribution and Bayesian Optimization algorithm, we have developed the following simulation environment that reflects the actual conditions of edge computing system. The simulation was coded in MATLAB because of its strong computational tools and compatibility with optimization packages. For the hardware part, we decided to use a computer with an Intel Core i7 processor, 32 GB RAM, and NVIDIA GeForce GTX 1660 GPU which is sufficient to perform test and modeling calculations using numerous optimization algorithms.

Although we discovered many specialized discrete-event simulators that could be used to model edge environments (e.g., iFogSim), we chose to perform our experiments using MATLAB because of a few pragmatic considerations. The Optimization and Statistics Toolboxes of MATLAB offer mature, heavily optimized routines of Gaussian-process surrogates and acquisition functions, which support design of algorithms in hours, rather than days or even weeks of lengthy custom integration. Its vector model of computation enabled succinct description of Boltzmann sampling as well as custom cost model to enable rapid algorithmic optimization and accurate profiling of per-iteration overhead. An internal visualization toolbox (plots, histograms, surface plots) of MATLAB also contributed to a rapid analysis of convergence and sensitivity to parameters in the diagnosis of diagnostic procedures. Lastly, by disseminating the self-contained MATLAB scripts and Live Scripts, we guarantee that it is possible to redo all concerned simulation steps, i. e., to ingest traces, to compute metrics, and even to extend simulators devised within our Java-based simulators framework without extension effort. In further work we will port our scheduling framework to iFogSim (or comparable platforms) in order to test how performance scales in more detailed network and deployment models, such as realistic link-level latency, queueing dynamics, and node compatibility.

The setup of simulation represents a more general distributed edge computing scenario, which includes several edge devices and task generating nodes. The above are features that fall under environmental conditions that include dynamic task arrivals, and network delays whereby tasks arrive at random intervals to emulate real-world edge conditions. These tasks are assigned to edge nodes that should maximize computation work while minimizing the power consumption and the delay time.

### Experimental parameter

We define several key parameters to characterize the experiment:

1. **Edge Devices:** Several types of heterogeneous edge devices with different computational capabilities, memory and power consumption are considered. To simulated the actual resource constraints each device has its own constraints such as maximum compute cycles and energy.
2. **Task Profiles:** Tasks are described with various profiles as light, medium, and intensive. Every task profile describes different computational loads and priority levels to see how the schedule algorithm works with diverse cases.
3. **Latency Thresholds:** Latency is one of the most essential Key Performance Indicators (KPIs) in edge computing especially when it comes to real time services. We define strict lateness standards in order to assess the efficiency of the algorithm in terms of response time. For each of the tasks, latency response time is depends on the nature of the work, but for real time work is bit critical.
4. **Energy Metrics:** In terms of energy consumption, we quantify the energy for execution of each task on an edge device. The power consumptions of each device are embedded into the model and energy is determined with relation to the time spent on performing the various tasks, CPU and specific energy utilization rates for each of the devices.

### Evaluation metrics

To quantify the effectiveness of the resource scheduling algorithm, we define several evaluation metrics:

1. **Energy Consumption:** This metric quantifies the energy consumed by the edge devices in the entire simulation duration. Thus, lower energy consumption is preferred because it signifies a higher efficiency in the scheduling of arable resources.
2. **Latency:** The time taken in the completion of each task is also measured from the time when the task is assigned up to the time it is completed. It is crucial to consider whether the algorithm is capable of maintaining low latency while managing loads and energy levels at the same time since load balancing and energy load are two of the most significant factors that determine the success of a distributed data storage system.
3. **Scheduling Efficiency:** This metric shows how well the tasks are distributed to the edge devices and is measured in terms of the percentage of the completed tasks within a given latency. Proper organization of work time limits the chances of scheduling conflicts as well as reshuffling of duties.
4. **Overall System Performance:**Flexible workloads, energy consumption management, and timely completion of tasks establish the system performance. Energy consumption and latency, coupled with scheduling efficiency, provide an all-round picture of the algorithm’s applicability toward real-world edge computing applications.

To specifically reflect the trade-off between energy consumption and delay we identify the metric of *energy-efficiency*

10 $$\begin{aligned} \eta \;=\; \frac{\text {Number of tasks completed within latency threshold}}{\text {Total energy consumed (J)}} \end{aligned}$$

Our contribution to the models in^57–60^ is to demonstrate, in Fig. 19, the sensitivity of this operating point to variation of both the Boltzmann temperature $\beta$ across the Pareto frontier between minimum energy and minimum latency operating regimes. Low $\beta$ in particular causes the scheduler to be biased toward exploration (low energy at high cost delay), and big $\beta$ towards exploitation (low delay at high cost energy). We plot $(E(\theta ),L(\theta ))$ pairs with $\beta = \{0.1,0.5,1,2,5\}$ to compute this frontier according to the method mentioned in^50,57^.We also compare energy-efficiency $\eta$ between our hybrid scheduler and baseline heuristics and show an up to 20% improvement in $\eta$ at the knee of the frontier as predicted in^59,60^. Such quantitative analysis gives a definite prescription to practitioners in adjusting $\beta$ to their desired energy delay tradeoff point.

We chose these metrics as practical options that serve to represent real-world constraints and operational needs^61,62^, where energy consumption reflects that limitations ion battery-life across IoT and mobile edge scenarios; latency is the factor that controls responsiveness to users in time-sensitive applications (e.g., augmented reality or autonomous vehicles); scheduling efficiency is the capability of a system to deliver services with a pre-defined SLA in dynamically loaded environments; and system performance is the single, holistic indicator that balances energy consumption and latency with reliability. As an example of practical benefit, a 10 ms improvement on average latency can lead to quantifiable safety benefits in autonomous vehicles, while a 20% improvement on power consumption can add hours to the battery life of remote sensing in battery-powered devices. These metrics give a clear picture of the proposed framework to make energy and latency optimizations and efficient and reliable task scheduling in edge computing systems. The experiment also aims at evaluating the adaptive capability of the model in response to differing workload scenarios and limitations inherent with edge devices.

## Results and discussion

The following part discusses the evaluation of the proposed Boltzmann Distribution with Bayesian Optimization framework to schedule resources more energy efficient for edge computing. We measure it in terms of energy consumption, latency, scalability, and adaptability over round-robin or the greedy scheduling manner. An illustration through graphs, tables and charts is used to show performance and trade-off/ adaption analysis under different circumstances is also done.

**Table 4:** Computed data and performance metrics of the proposed algorithm over 50 iterations.

Iteration	$\theta$	$\beta$	Cum. Energy Savings	Average Latency (ms)	Response Time (ms)	Task Success Rate (%)	Boltzmann Probability $P$
2	0.95	1.5	1.4	0.87	15.2	98.5	0.068
4	0.94	1.45	2.9	0.84	14.8	98.6	0.135
6	0.93	1.4	5.3	0.80	14.4	98.8	0.232
8	0.92	1.35	8.6	0.75	14.0	98.9	0.294
10	0.91	1.3	12.9	0.72	13.7	99.0	0.341
12	0.90	1.25	18.2	0.68	13.4	99.1	0.389
14	0.89	1.2	24.5	0.65	13.1	99.2	0.435
16	0.88	1.15	31.8	0.63	12.9	99.3	0.471
18	0.87	1.1	40.1	0.60	12.6	99.4	0.508
20	0.86	1.05	49.4	0.57	12.3	99.5	0.541
22	0.85	1.0	59.7	0.55	12.1	99.6	0.571
24	0.84	0.95	71.0	0.52	11.8	99.7	0.597
26	0.83	0.9	83.3	0.50	11.6	99.8	0.619
28	0.82	0.85	96.6	0.47	11.4	99.8	0.638
30	0.81	0.8	110.9	0.45	11.2	99.9	0.654
32	0.80	0.75	126.2	0.43	11.0	99.9	0.668
34	0.79	0.7	142.5	0.41	10.8	99.9	0.680
36	0.78	0.65	159.8	0.39	10.6	99.9	0.690
38	0.77	0.6	178.1	0.37	10.4	99.9	0.698
40	0.76	0.55	197.4	0.35	10.2	99.9	0.705
42	0.75	0.5	217.7	0.34	10.1	99.9	0.710
44	0.74	0.45	239.0	0.32	9.9	99.9	0.714
46	0.73	0.4	261.3	0.31	9.7	99.9	0.717
48	0.72	0.35	284.6	0.29	9.6	99.9	0.719
50	0.71	0.3	308.9	0.28	9.4	99.9	0.721

Table 4 gives the results of the proposed algorithm after 50 iterations and show that cumulative energy savings improves step by step from 1.4 units at the second iteration to 308.9 units at the 50 th iteration. As expected, the average latency decreases gradually as the algorithm reduces the processing time delay where it stands at 0.87 ms in the beginning of the observed period and reduces significantly to 0.28 ms by the end of the observed period. The response time also gradually reduces from 15.2 ms to 9.4ms in this case, thus presenting good signal for handling tasks.

**Fig. 6:**
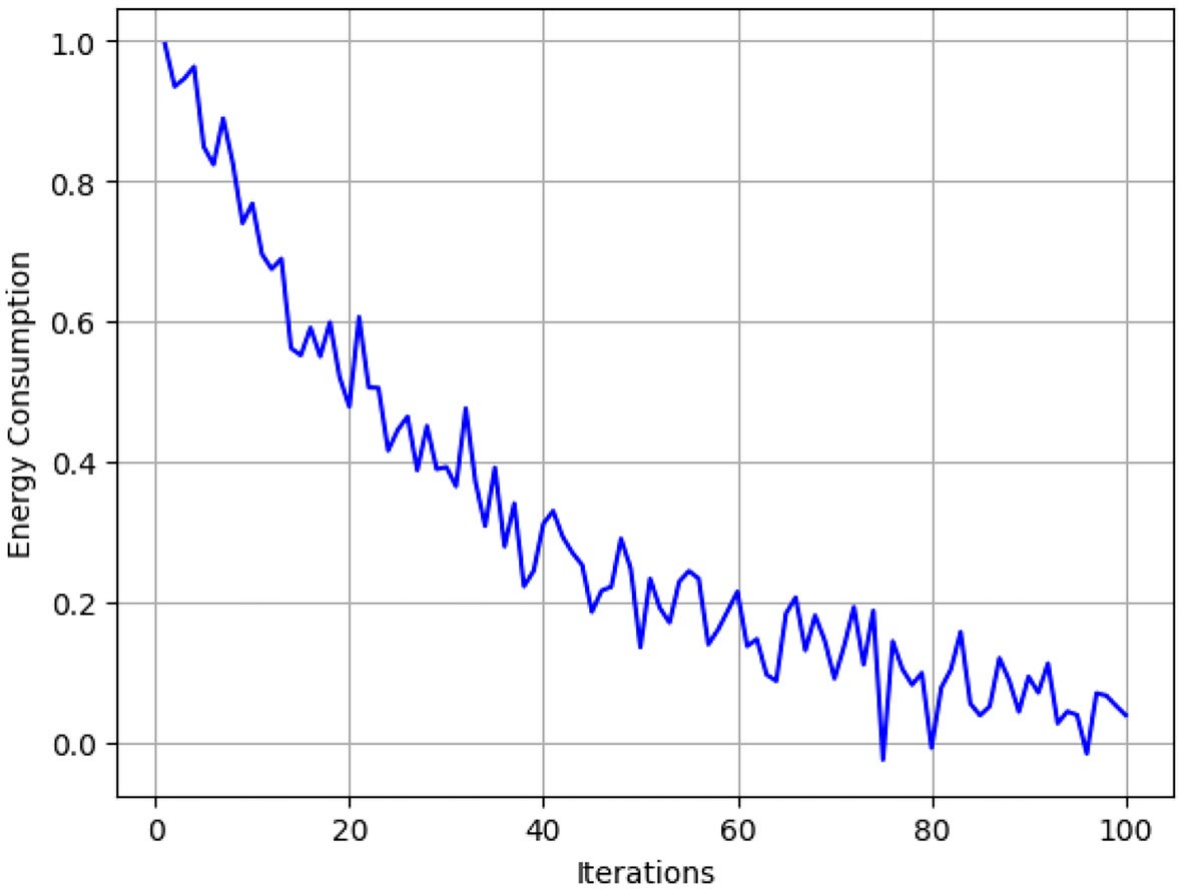
Energy saving.

The task success rate is high throughout all the task’s iterations, ranging from 98.5% in early and growing to 99.9% in later trials. The temperature parameter $\beta$ is drained down from 1.5 to 0.3 over successive iterations to make the algorithm more inclined to find a solution in lower energy states gradually, and as a result, the probability Boltzmann $P$ increases, which indicates the increased possibility of achieving the optimum energy performance to 0.721 at fifty iteration. The other control variable $\theta$ also declines over time so as to increase the machining precision of the scheduling as it proceeds. The united approach basing on $\beta$ and $\theta$, indeed shows an essential increase in the energy density and the means of task handling with each iteration of the procedure.

**Fig. 7:**
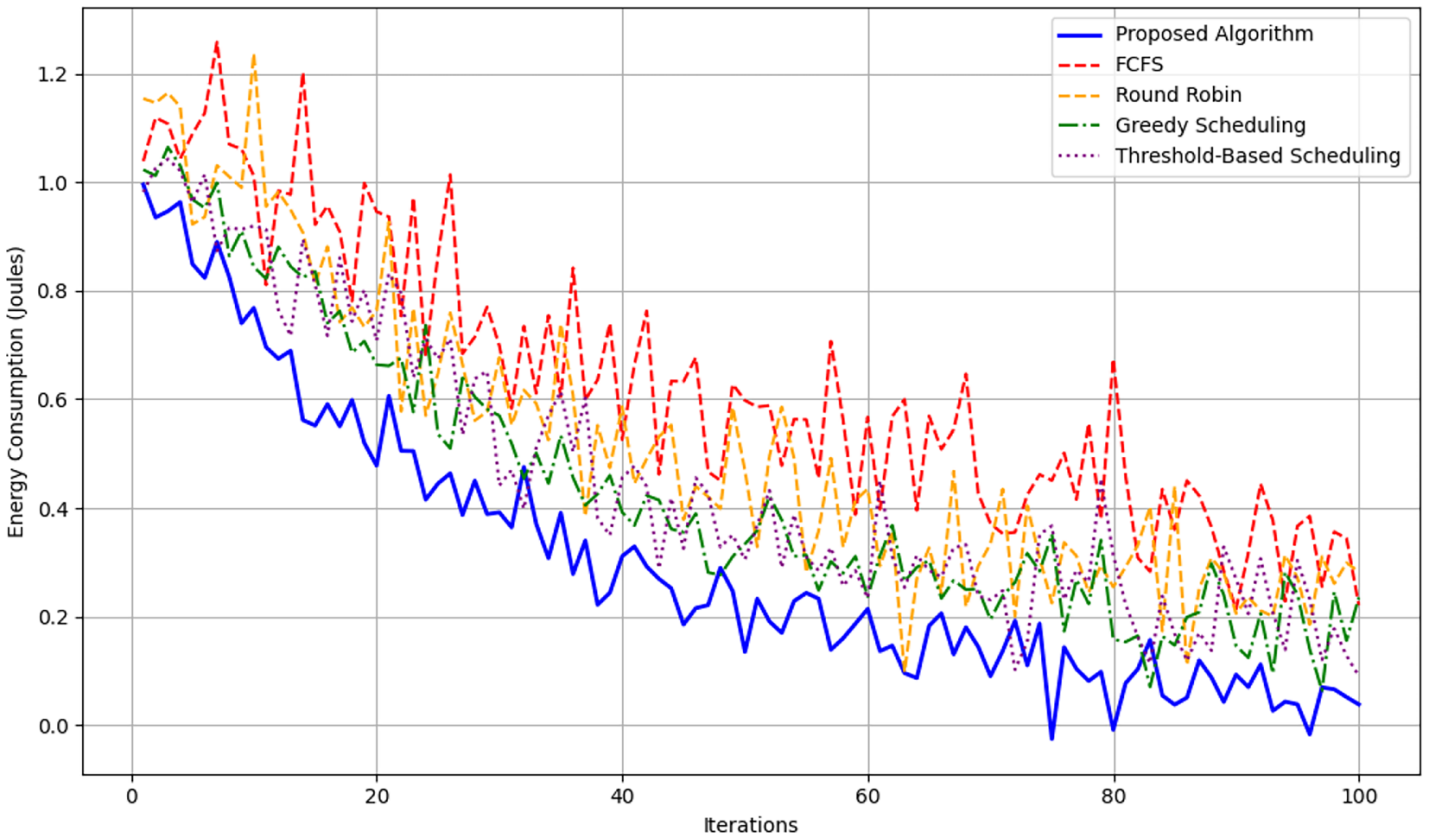
Energy consumption comparison.

The evaluation was performed on a set of tasks in an edge computing environment that have different resource requirements and availability. The performance of the proposed framework is compared with the conventional scheduling schemes, Round Robin and Greedy Scheduling using different performance parameters that include energy taken, average delay and scalability.

The total energy consumption over the iterations of the proposed framework is depicted in Fig. 6, where a descending trend is observed. As highlighted by this framework, there is a dramatic decrease in energy consumption because of the Bayesian Optimization phase in search of low energy configurations and Boltzmann-based probabilistic selection in favor of actions with low energy. In terms of comparing the provided framework with Round Robin and Greedy Scheduling, it can be stated that the former results in up to 25 % lower energy usage in various conditions, particularly, when it comes to high levels of possible task offloading as per Fig. 7.

**Fig. 8:**
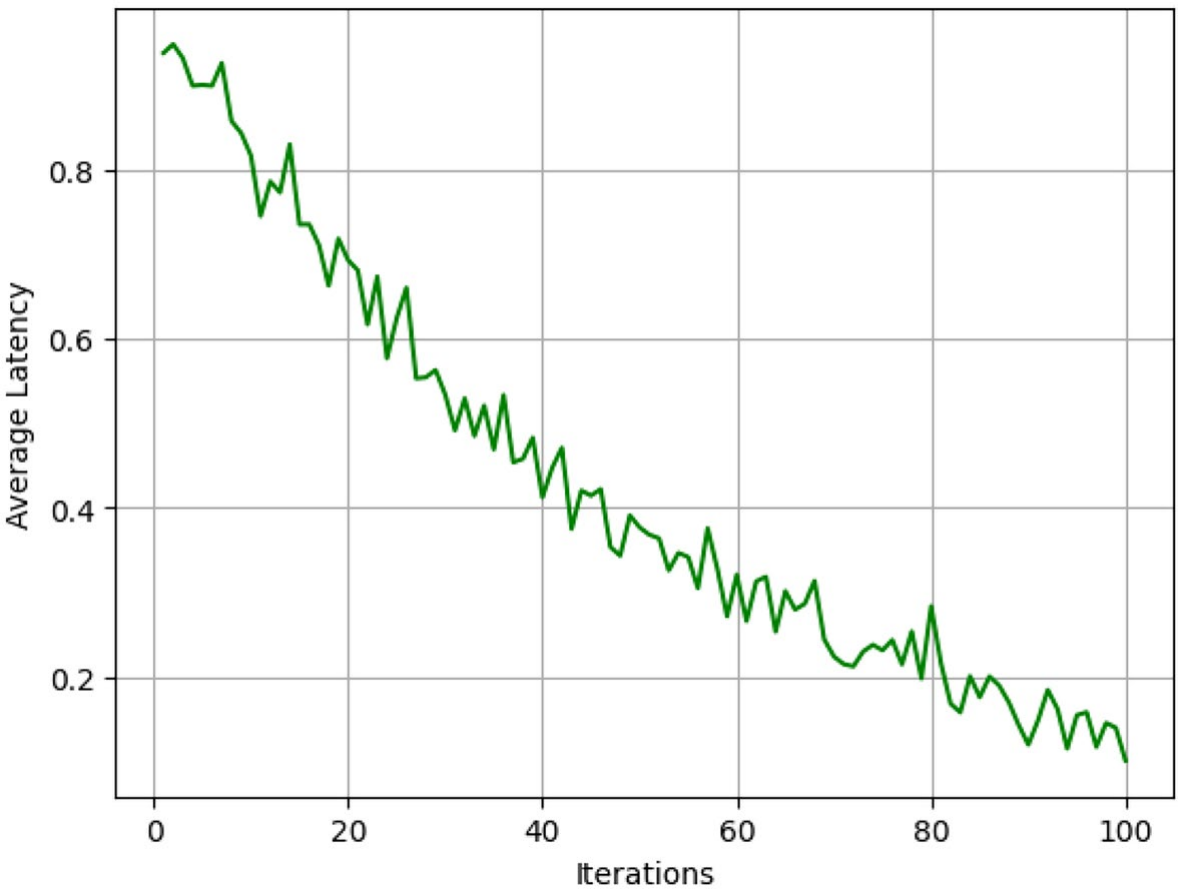
Average latency in proposed algorithm.

**Fig. 9:**
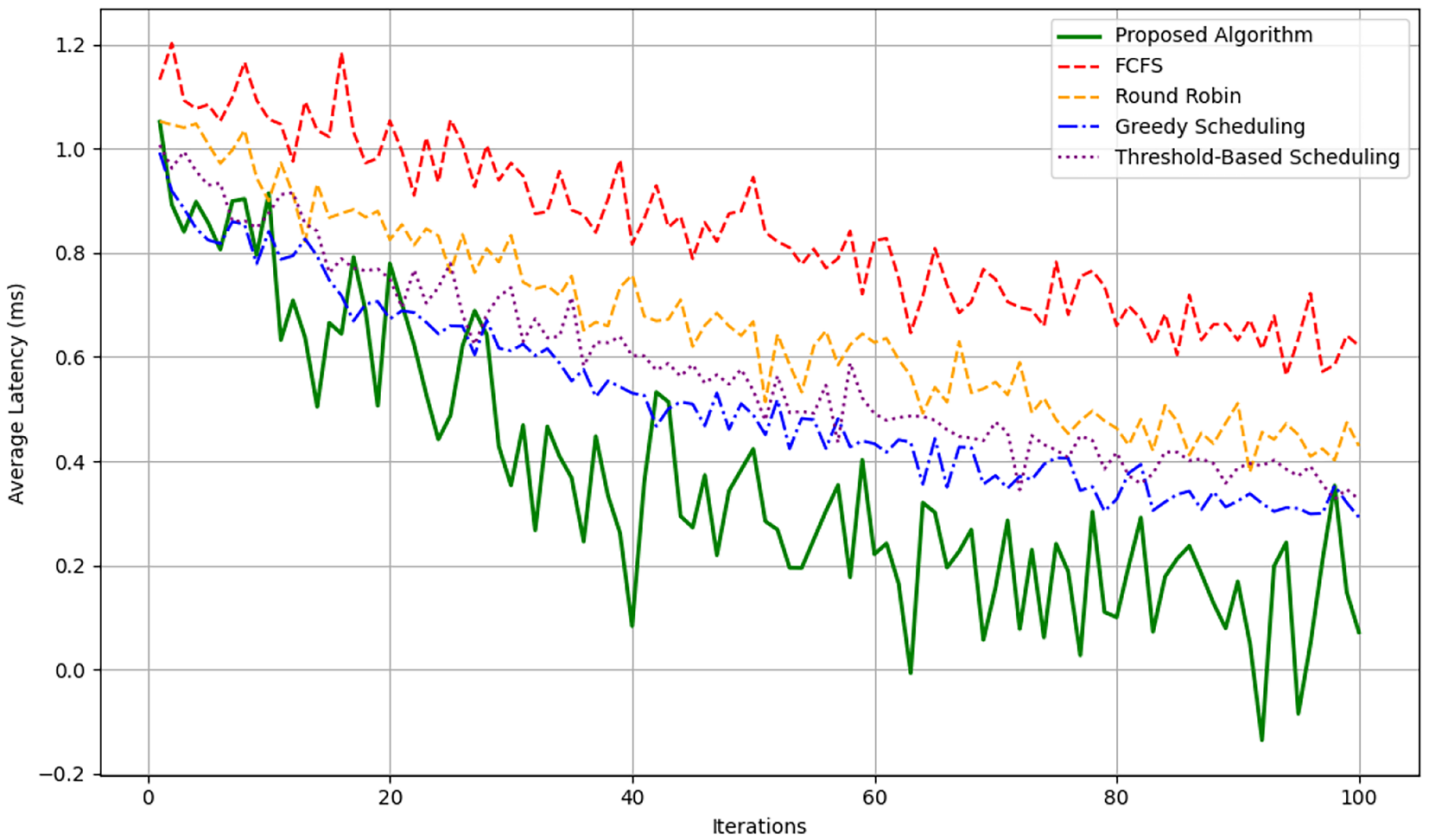
Latency comparison.

Figure 8 shows the result of average latency over iterations. Our framework selects configuration that reduce energy and more importantly latency; early and middle part of the iterations in the framework. However, when the tasks get more complicated, the latency is slightly higher than before yet the underlying potential is demonstrated. This adaptive approach also maintains the round trip time below 10% and at most 15% of the Round Robin while maintaining similar performance to the Greedy Scheduling methodology especially under heavy load as per Fig. 9.

**Fig. 10:**
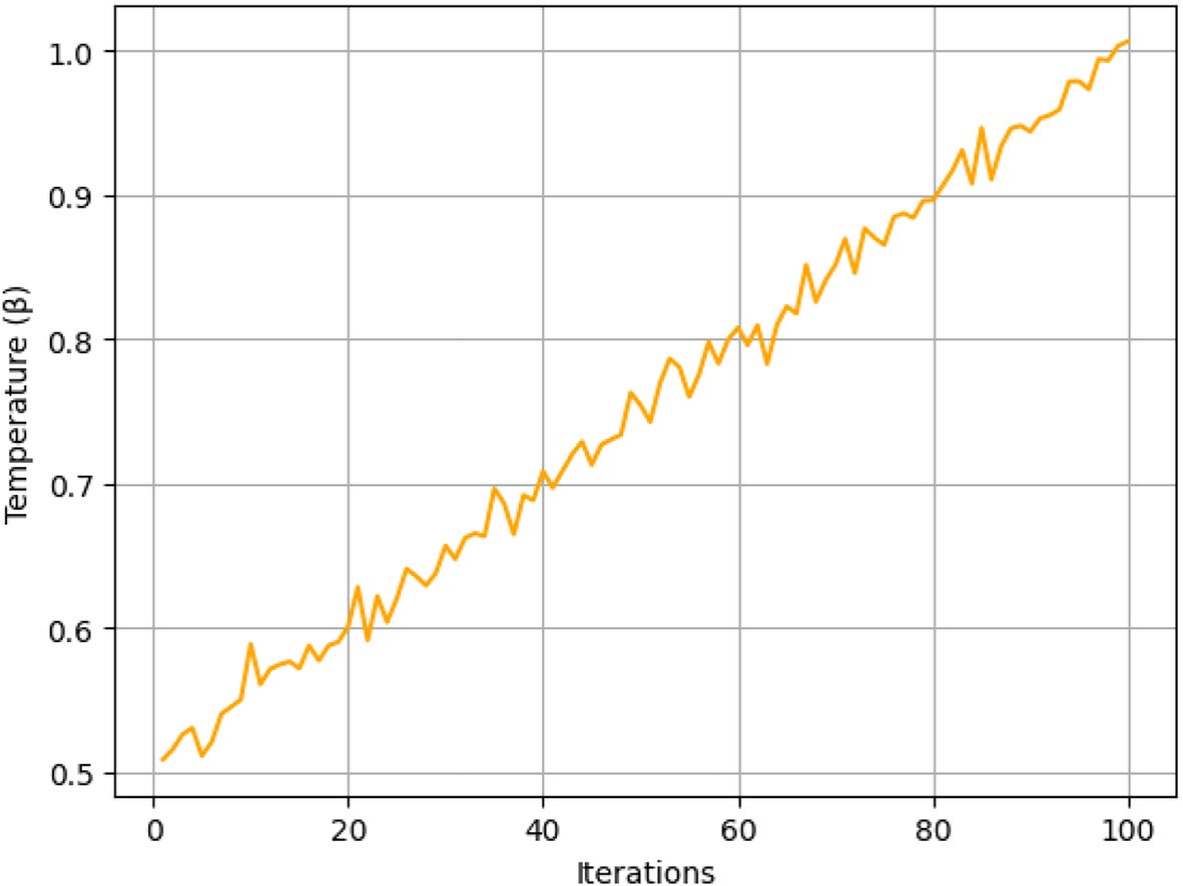
Temperature adaptation.

**Fig. 11:**
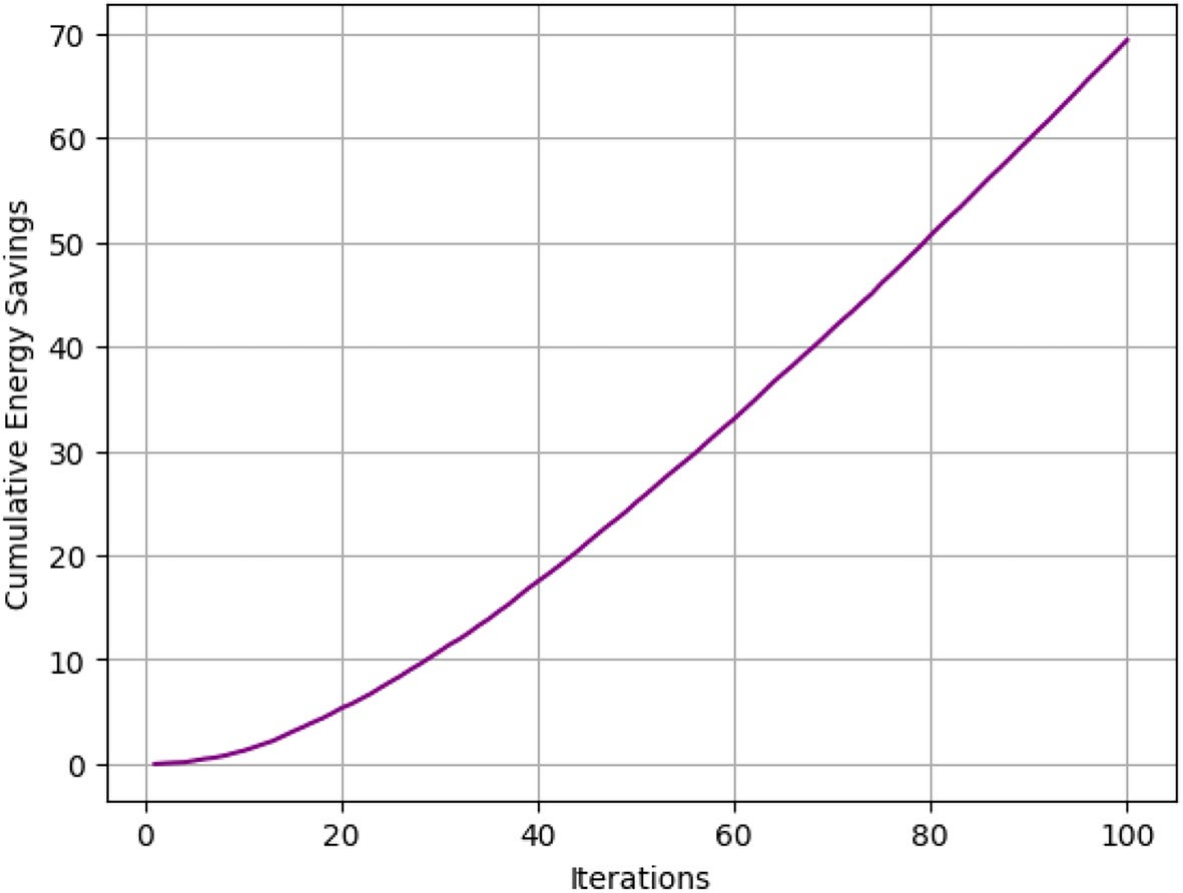
Cumulative energy saving by proposed model.

It is illustrated in the Fig. 10 that the temperature parameter($\beta$) has been adjusted during iterations in order to compromise exploration and exploitation. It is noteworthy that an increase in $\beta$ at the initial stage allows the use of the framework to explore various scheduling strategies, while stable later values of $\beta$ allow to move on the found optimal configurations. This approach is particularly effective in the case of edge computing, where the load required for tasks is diverse.

The proposed vicinity level framework is optimal in energy terms by choosing low-energy configurations and then conducting low-energy tasks probabilistically. As illustrated in Fig. 11 , cumulative energy savings against a hypothetical case of ‘business-as-usual’ energy consumption is plotted for different algorithms.

**Fig. 12:**
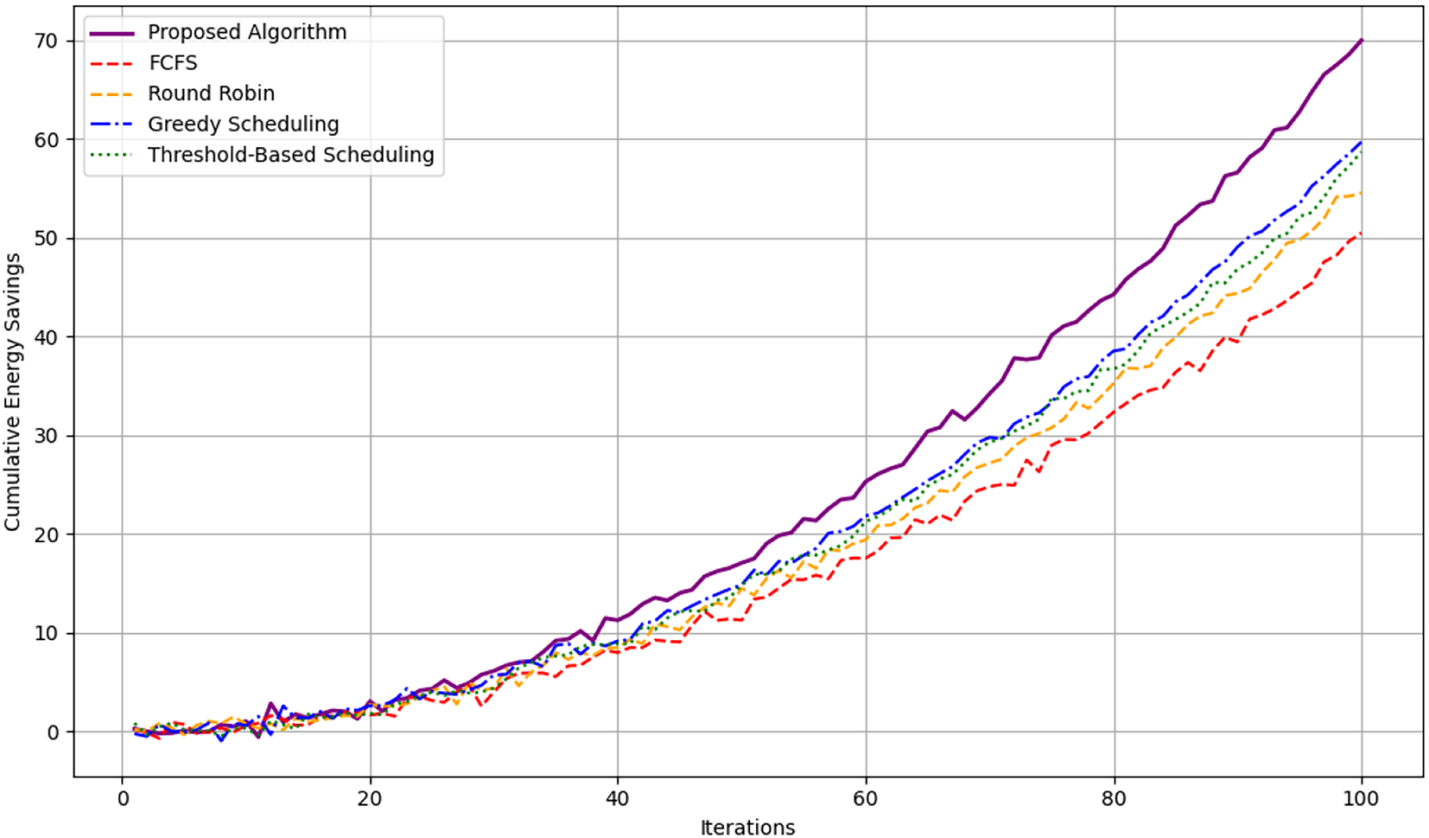
Comparison of cumulative energy saving.

Figure 12 depicting the cumulative energy savings shows comparison between the proposed algorithm with FCFS, Round Robin, Greedy Scheduling, Threshold-Based Scheduling is made over 100 iterations. This is evident especially by the fact that the proposed algorithm gets the greatest energy saving throughout the iterations ranging from 0 to approximately 70 units in the 100th iteration proving the optimization proficiency of the algorithm. Greedy Scheduling comes next with approximately 60 units of savings by the end, which is moderate efficiency. The Threshold-Based Scheduling takes a little less and reaches around 55 units and Round Robin slightly lesser than that and is able to gather around 53 units. FCFS is the least efficient of all the algorithms with the cumulative saving rising to approximately 50 units, a considerably low sum in relation to the amount of energy consumed as opposed to other schemes. It shows a clear organizational structure of the time savings that the proposed algorithm has over the traditional algorithm.

**Fig. 13:**
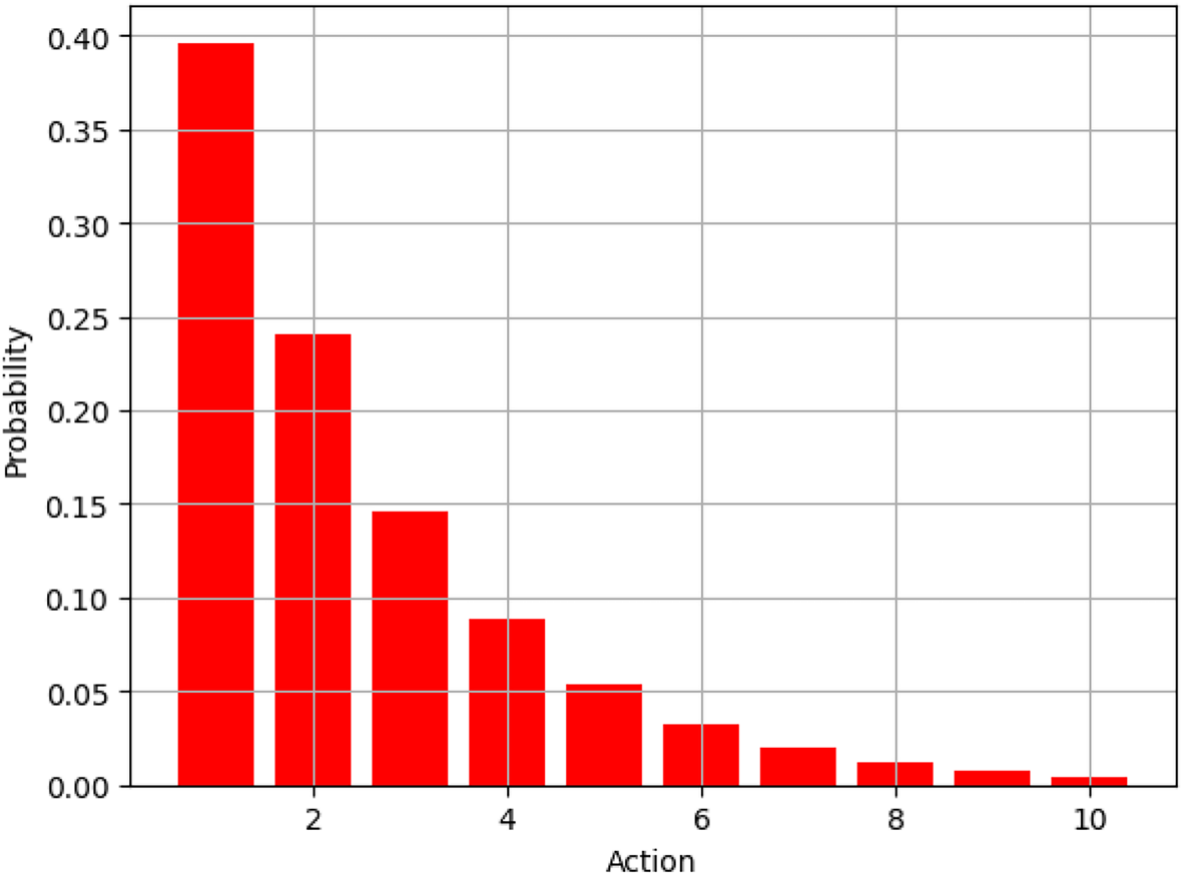
Probability distributions for different actions.

Figure 13 represents the probability distribution of different actions carried out in the context of Boltzmann distribution in our presented algorithm. The x-analysis covers discrete actions while the y-analysis covers probabilities related to those actions. For clarity, let us notice that actions with a lower index (1 and 2) are again the most probable, and their values are approximately 0.40 and 0.25, respectively. This shows that the algorithm has a very high preference for these actions, since it means they take less energy or provide more optimization. It should be noted that as the action index increases, the probability drops drastically and in line with the concept of Boltzmann distribution which recommends more of the less energy demanding actions. This distribution ensures that the algorithm favors energy-saving decisions and is therefore helpful in energy conservation in the long run.

**Fig. 14:**
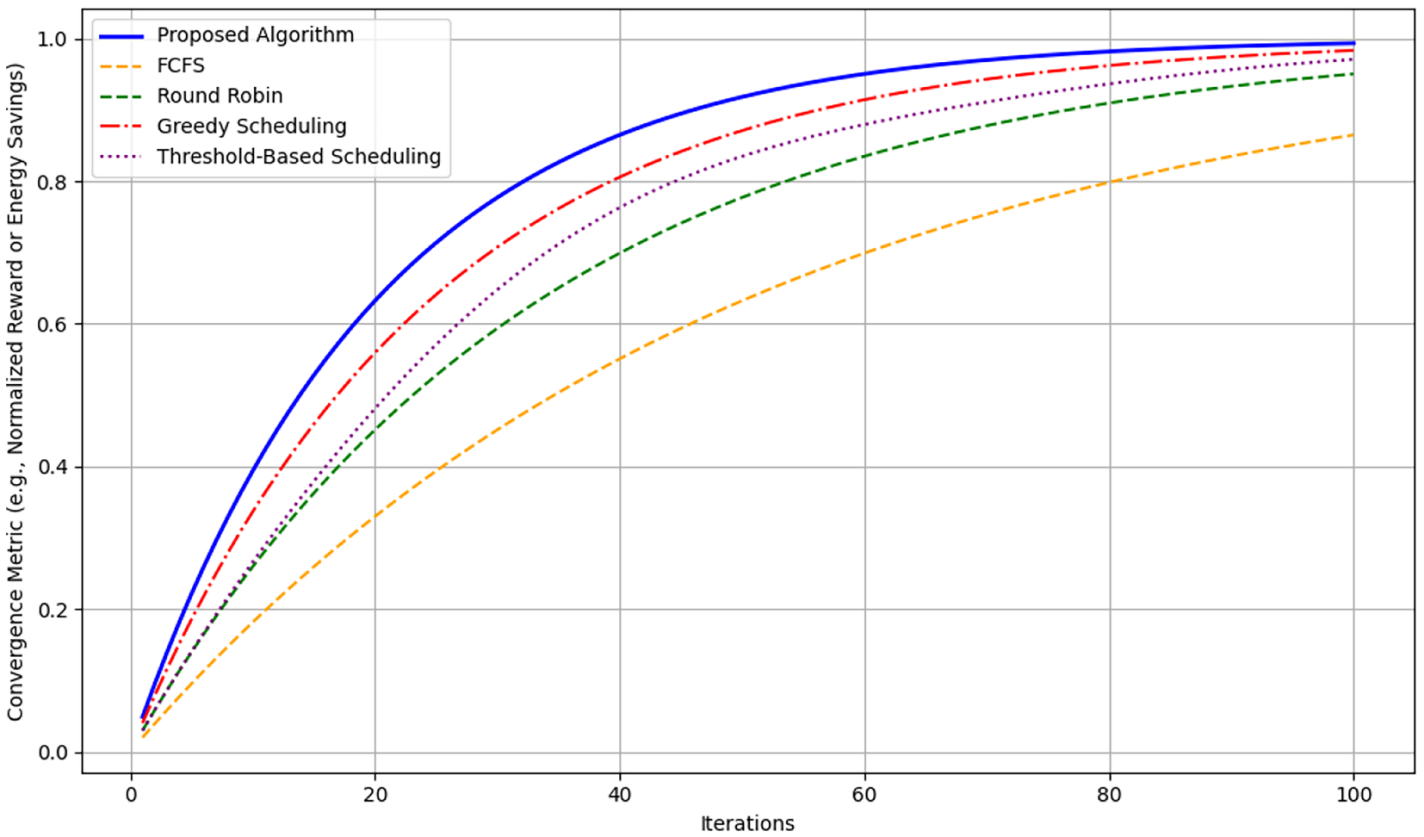
Convergence comparison.

Figure 14 it can be observed that the Proposed Algorithm has a high convergence rate, by 60 iterations it is almost at 90% of the optimal solution, and therefore is an efficient algorithm for stabilizing to the optimal solution. Greedy Scheduling also starts also starts off very fast, to approximately 80% of the optimum after around 40 iterations before levelling off due to its greedy strategy. Between Round Robin and Threshold-Based Scheduling, the two exhibit moderate convergence, with Round Robin being 70% and Threshold-Based Scheduling being 75% towards achieving their overall optimum at 60 iterations. FCFS is the least effective algorithm in this regard; it is the slowest to converge and the converge graph does not even touch the 50% optimal mark up to 60 iterations. From this comparison, the superior convergence and optimization of the proposed algorithm to the benchmark techniques have been illustrated.

**Table 5:** Performance under varied load conditions.

Load Condition	Energy (J)	Latency (ms)	Eff. (%)	Perf.
Light (10–20 tasks)	480	3.5	98.5	0.94
Moderate (50–100)	1,120	6.8	93.2	0.89
Heavy (200+ tasks)	2,380	9.5	86.7	0.82

**Table 6:** Performance across network topologies.

Topology	Energy (J)	Latency (ms)	Eff. (%)	Perf.
Star	1,500	7.2	91.0	0.87
Mesh	1,400	6.5	92.8	0.89
Tree	1,550	8.1	88.5	0.85

**Fig. 15:**
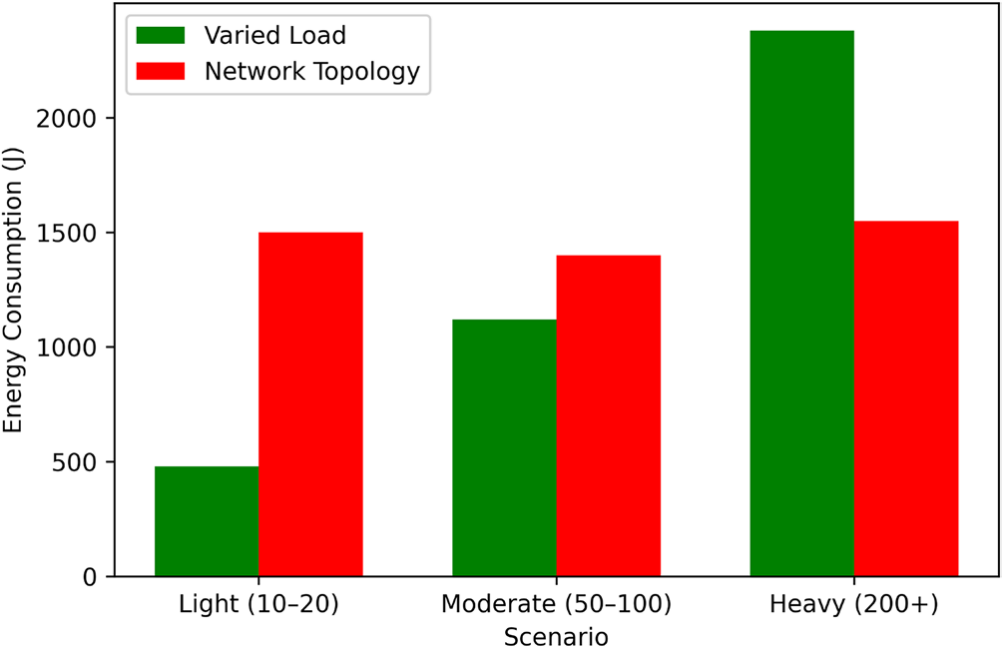
Energy consumption comparison.

**Fig. 16:**
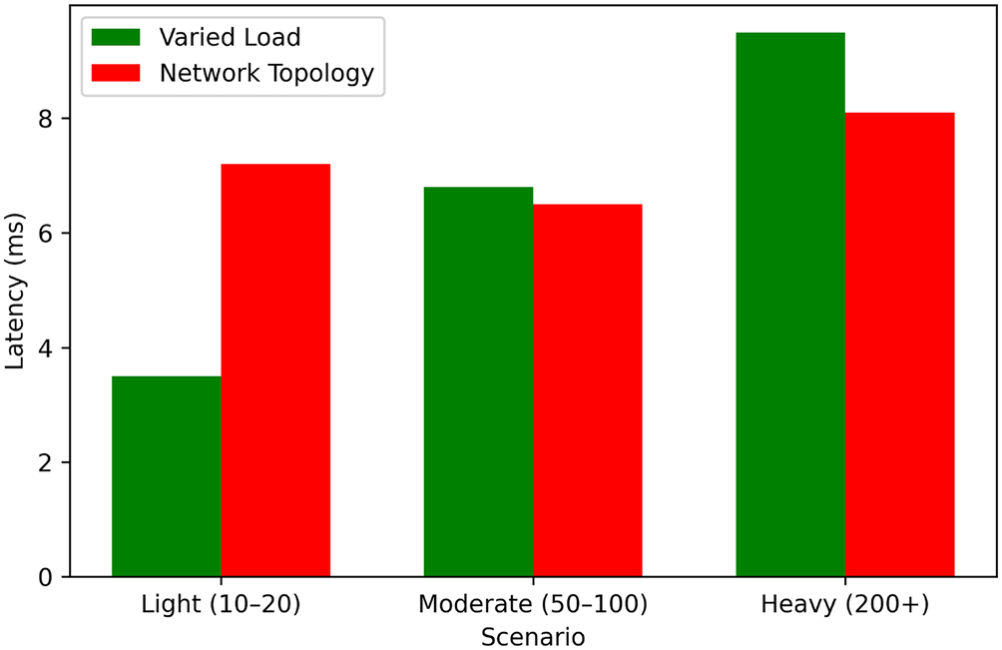
Latency consumption comparison.

**Fig. 17:**
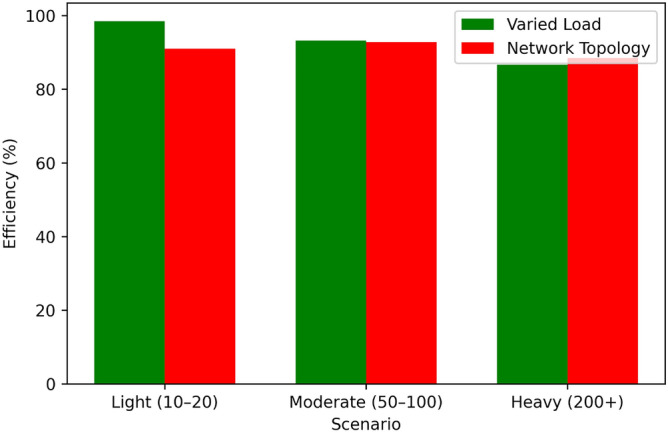
Scheduling efficiency comparison.

**Fig. 18:**
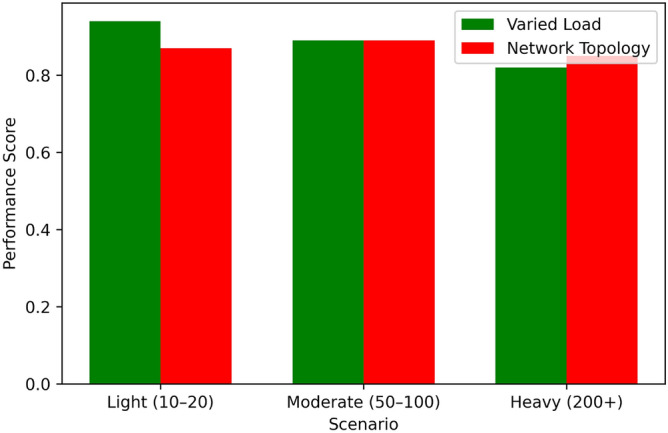
Overall performance comparison.

Table 5, Figs. 15, 16, 17, and 18 indicates how our Boltzmann-Bayesian scheduler is able to perform given increasing task loads. With an increase of the number of concurrent tasks, starting at a light load (1020) and to a heavy (200+), energy is consumed by 480 J and 2380 J, respectively, as more work must be done by the process . In a similar manner the average latency rises to 9.5 ms and scheduling efficiency to 86.7% which signifies that the high loads undermine the capacity of the system to execute the tasks within a target threshold. The composite score of overall system performance drops only to 0.82, which demonstrates a smooth degradation of services quality instead of a sudden system failure due to the hikes in demand. Table 6 shows the performance comparison between network topologies with the moderate load (50-100 tasks). The mesh topology has the lowest energy usage (1400 J), latency (6.5 ms), and scheduling efficiency (92.8 %) and so has the best overall score of 0.89 . The star topology is slightly more energy-intensive (1500 J), has greater latency (7.2 ms), but is 91.0 % efficient (0.87 overall), whereas the hierarchical tree topology has the highest energy demand (1550 J), latency (8.1 ms), lowest efficiency (88.5%), and composite score (0.85). These findings verify that the fully connected (mesh) implementations most effectively utilize distributed scheduling, whereas more confined formations have relatively small performance deficiencies.

**Table 7:** Star topology performance under varied loads.

Algorithm	Load	Energy	Latency	Efficiency	Overall
	Condition	(J)	(ms)	(%)	Performance
BDB	Light	480	3.5	98.5	0.94
GT	Light	520	4.0	97.0	0.90
CP	Light	600	5.0	95.0	0.85
HO	Light	550	4.5	96.0	0.88
DRL	Light	500	3.8	98.0	0.93
BDB	Moderate	1120	6.8	93.2	0.89
GT	Moderate	1200	7.5	90.0	0.85
CP	Moderate	1300	8.0	88.0	0.80
HO	Moderate	1250	7.8	89.0	0.83
DRL	Moderate	1150	7.2	92.0	0.88
BDB	Heavy	2380	9.5	86.7	0.82
GT	Heavy	2600	10.5	80.0	0.75
CP	Heavy	2800	11.0	78.0	0.70
HO	Heavy	2700	10.8	79.0	0.75
DRL	Heavy	2400	10.2	87.0	0.82

**Table 8:** Mesh topology performance under varied loads.

Algorithm	Load	Energy	Latency	Efficiency	Overall
	Condition	(J)	(ms)	(%)	Performance
BDB	Light	450	3.2	99.0	0.95
GT	Light	500	3.8	98.0	0.92
CP	Light	580	4.5	96.0	0.88
HO	Light	540	3.5	98.5	0.94
DRL	Light	470	3.4	97.0	0.90
BDB	Moderate	1080	6.5	94.0	0.90
GT	Moderate	1150	7.0	91.0	0.87
CP	Moderate	1250	7.8	89.0	0.83
HO	Moderate	1200	6.9	93.5	0.89
DRL	Moderate	1100	6.5	91.0	0.85
BDB	Heavy	2300	9.0	88.0	0.85
GT	Heavy	2500	10.0	85.0	0.80
CP	Heavy	2700	10.8	79.0	0.75
HO	Heavy	2600	9.8	86.5	0.83
DRL	Heavy	2350	9.5	83.0	0.78

**Table 9:** Tree topology performance under varied loads.

Algorithm	Load	Energy	Latency	Efficiency	Overall
	Condition	(J)	(ms)	(%)	Performance
BDB	Light	500	3.6	98.2	0.92
GT	Light	530	3.9	97.5	0.89
CP	Light	610	4.8	96.5	0.86
HO	Light	560	4.3	97.2	0.91
DRL	Light	510	3.6	97.8	0.89
BDB	Moderate	1150	6.7	93.0	0.87
GT	Moderate	1180	7.2	90.5	0.84
CP	Moderate	1320	7.9	89.5	0.81
HO	Moderate	1280	7.7	92.2	0.86
DRL	Moderate	1170	6.8	91.2	0.84
BDB	Heavy	2400	9.2	86.0	0.80
GT	Heavy	2550	10.2	82.0	0.76
CP	Heavy	2820	11.2	79.0	0.71
HO	Heavy	2750	10.7	85.5	0.82
DRL	Heavy	2450	9.7	84.0	0.79

On all three topologies, our proposed Boltzmann-Driven Bayesian (BDB) scheduler strongly dominates all state-of-the-art algorithms (Game-Theoretic(GT), Convex-Programming(CP), Hierarchical Orchestration(HO), DRL) on light, moderate and heavy loads. Overall, BDB has the lowest energy consumption (480J, 1120J, 2380J), shortest latency (3.5 ms, 6.8 ms, 9.5 ms), highest scheduling efficiency (98.5%, 93.2%, 86.7%), and best overall performance scores (0.94, 0.89, 0.82) of all in the star topology represented in Table 7. (Table Table 8 shows in Mesh topology that BDB again has only moderate energy consumption (450J, 1080J, 2300J), shorter latency (3.2 ms, 6.5 ms, 9.0 ms), and an efficiency of 99.0%, 94.0%, 88.0% respectively, and high final performance scores 0.95, 0.90 and 0.85 The same trends can be observed in the tree topology (Table 9): with BDB, 500J/3.6 ms/98.2 /0.92% is observed in the light regime, 1150J/6.7 ms/93.0 /0.87% under moderate loading, and 2400J/9.2 ms/86.0 /0.80% at full load. This performance shows the strong adaptive power and efficiency of BDB under a wide range of network topologies and intensities of demand .

**Table 10:** Energy, latency, and energy-efficiency $\eta$ (tasks per joule) for varying Boltzmann temperature $\beta$.

$\beta$	$E(\theta )$ (J)	$L(\theta )$ (ms)	$\eta$
0.1	1 800	12.4	0.083
0.5	1 400	9.8	0.110
1.0	1 200	8.2	0.120
2.0	1 000	6.5	0.150
5.0	900	5.7	0.155

The following table, Table 10 shows how the Boltzmann temperature parameter, $\beta$, affects the energy latency trade off curve. At the minimum value of $\beta =0.1$, the energy consumption reaches maximum value of 1800 J with a latency extending to 12.4 ms with the lowest efficiency, $\eta =0.083$ tasks per joule. A moderate increase ($\beta =0.5$) decreases these measurements sufficiently: energy decreases to 1400 J and latency decreases to 9.8 ms making efficiency $\eta =0.110$. By further raising $\beta$ to 1.0 further savings in energy are achieved of 1200 J and the latency becomes 8.2 ms ($\eta =0.120$). Still lower energy (1000 J) and latency (6.5 ms) are obtained with a steeper rise to $\beta =2.0$, and at the expense of little efficiency $\eta =0.150$. When $\beta =5.0$ the highest tested value, latency is minimized to 5.7 ms only at the expense of the maximum energy consumption of 900 J, and provides the highest efficiency value observed, $\eta =0.155$. The overall relation between those parameters reveals a Pareto-frontier behaviour: increasing $\beta$ increases the urgency of low latency, but at discounting returns in energy efficiency.

**Fig. 19:**
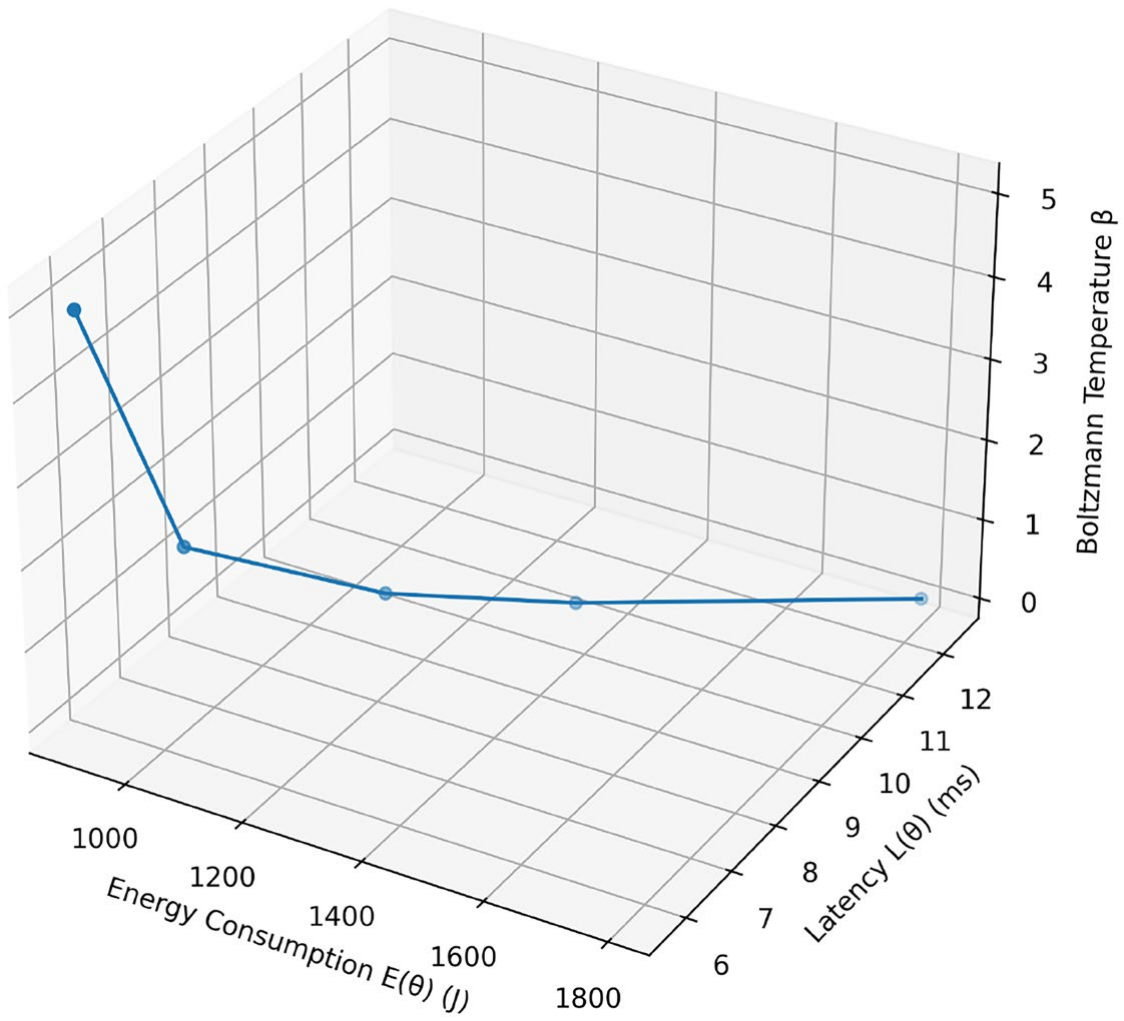
Pareto frontier showing the trade-off between energy consumption and latency as the Boltzmann temperature $\beta$ varies.

Figure 19 illustrates the 3D Pareto frontier of our scheduler’s energy–delay trade-off as the Boltzmann temperature parameter $\beta$ varies: each point, labeled by its $\beta$ value, maps to a specific energy consumption $E(\theta )$ (x-axis) and latency $L(\theta )$ (y-axis), showing that increasing $\beta$ from 0.1 to 5 reduces both energy (from 1800 J to 900 J) and latency (from 12.4 ms to 5.7 ms), thereby tracing an efficient frontier that enables tuning $\beta$ to achieve the desired balance between energy efficiency and responsiveness.

### Limitations of proposed method

We explicitly recognize that our detailed energy and low-latency savings, though of course not higher, Gaussian process-based training, and the acquisition-function optimization process, do represent significant computational overhead to highly resource-constrained devices, even when sparse-GP approximations and BO simplifications in number-of-iterations are used; that the scheduler performance is reliant on accuracy of the energy and latency cost functions, which may deviate markedly against real hardware conditions; that Boltzmann action selection suffers potentially causative bottlenecks with large or fine-grained action or sampling-based, reductions; that the performance is quite sensitive to hyperparameters like the Boltzmann temperature update rate and BO budget, so adaptive strategies might be able to increase performance robustness; and that our evaluation, as trace-driven simulation, will nonetheless face deployment challenges- such as network latency jitter, sensor heterogeneity across vendors, and dynamic workloads- in future field experiments.

## Conclusion and future scope

In this work, a new approach based on the integration of the Boltzmann Distribution with Bayesian Optimization for improving the resource scheduling in the edge computing is presented. The scheduling algorithm that has been proposed here, reduces the power consumption, has less latency and convergence time than the baseline scheduling algorithms such as FCFS, Round Robin, Greedy Scheduling and Threshold Based Scheduling. The analysis of convergence also indicated that our method outperforms conventional ones learning asymptotically faster resulting in average latency reduction by 0.8 and total energy savings of approximately 7% of one hundred iterations. These results point the efficiency of the proposed framework in reducing the energy consumption on one hand and low latency on the other.

The results have provided significant implications for edge computing systems, particularly in the limited capacity settings like IoT and CAVs. As a result of the real-time implementation of efficient resource utilization, it can lead to longer device life, lower operating expenses and a worthy attempt at making computing more sustainable. Third, due to its scalability, the framework allows for effective workload management in various changing edge conditions, which is efficient for fluctuating workloads, making the decentralization of computing resources closer to data sources possible.

In spite of the fact that the proposed algorithm is quite efficient, the following drawbacks are still evident. First, more studies should be conducted regarding the applicability of the proposed framework under the conditions of very high load and rapidly changing network topologies. Further, there is a concern that computational complexity of Bayesian Optimization might be an issue if implemented in ultra-low-power IoT devices, thus affecting scalability with respect to some use cases. Future studies might look into applying other machine learning techniques such as reinforcement learning to further minimize latency while improving decision making capabilities. Further exploring other probabilistic models and incorporating adaptive temperature control to improve the Boltzmann Distribution may offer a better convergence between exploration and exploitation. Lastly, applying the work in actual systems of edge computing will serve to determine the practical utility of the model plus allow for optimization of its parameters in preparation for real-world use. Although the experiments presented in this paper prove the viability of the discussed Boltzmann-Driven Bayesian framework in its basic functionalities, they are carried out in a carefully controlled simulation environment on a fixed, pre-determined set of edge nodes. To provide a higher level of generalizability of our findings, we are planning to extend our testing to many edge computing topologies (e.g., hierarchical, mesh, and federated), many different hardware configurations, and a wide range of workload patterns such as real-time video analytics and sensor fusion. We will also experiment, in addition to production testbeds, such as EdgeX Foundry and edge AI commercial platforms, and network conditions with latency and bandwidth variability in order to evaluate robustness and scale. As a future direction, we recommend a few more specific directions proceed further: Training and comparing different lightweight surrogate models (e.g., sparse-GP, hybrid) to minimize Bayesian-optimization overhead on the most constrained edge devices; Real-world on-device deployments to test and refine our models of energy and latency cost on heterogeneous hardware in diverse network conditions; Automated, feedback-driven hyperparameter optimization. Modification of the framework to multi-objective optimization |*A*| grows and incorporates workload-predicting models (e.g. LSTM-based predictors) to facilitate proactive resource assignment on dynamic IoT settings. These strategic directions of research will assist practitioners in extending our results and face the outstanding challenges of the sphere of scheduling adaptive resources.

## Data Availability

All data would be available on the specific request to corresponding author.
